# *Mycobacterium smegmatis* Bacteria Expressing *Mycobacterium tuberculosis*-Specific Rv1954A Induce Macrophage Activation and Modulate the Immune Response

**DOI:** 10.3389/fcimb.2020.564565

**Published:** 2020-10-09

**Authors:** Simran Kaur Arora, Nilofer Naqvi, Anwar Alam, Javeed Ahmad, Basma Saud Alsati, Javaid Ahmad Sheikh, Prabin Kumar, Dipendra Kumar Mitra, Syed Asad Rahman, Seyed Ehtesham Hasnain, Nasreen Zafar Ehtesham

**Affiliations:** ^1^Indian Council of Medical Research (ICMR)-National Institute of Pathology, Safdarjung Hospital Campus, New Delhi, India; ^2^Institute of Molecular Medicine, Jamia Hamdard, New Delhi, India; ^3^Department of Biotechnology, Jamia Hamdard, New Delhi, India; ^4^Department of Transplant Immunology and Immunogenetics, All India Institute of Medical Sciences, New Delhi, India; ^5^BioInception Pvt. Ltd., Chelmsford, United Kingdom; ^6^Dr. Reddy's Institute of Life Sciences, University of Hyderabad Campus, Hyderabad, India

**Keywords:** host-pathogen interface, immune modulation, oxidative stress, signature protein, Th1 response

## Abstract

*Mycobacterium tuberculosis* (*M. tb*), the intracellular pathogen causing tuberculosis, has developed mechanisms that endow infectivity and allow it to modulate host immune response for its survival. Genomic and proteomic analyses of non-pathogenic and pathogenic mycobacteria showed presence of genes and proteins that are specific to *M. tb*. *In silico* studies predicted that *M.tb* Rv1954A is a hypothetical secretory protein that exhibits intrinsically disordered regions and possess B cell/T cell epitopes. Treatment of macrophages with Rv1954A led to TLR4-mediated activation with concomitant increase in secretion of pro-inflammatory cytokines, IL-12 and TNF-α. *In vitro* studies showed that rRv1954A protein or Rv1954A knock-in *M. smegmatis* (Ms_Rv1954A) activates macrophages by enhancing the expression of CD80 and CD86. An upregulation in the expression of CD40 and MHC I/II was noted in the presence of Rv1954A, pointing to its role in enhancing the association of APCs with T cells and in the modulation of antigen presentation, respectively. Ms_Rv1954A showed increased infectivity, induction of ROS and RNS, and apoptosis in RAW264.7 macrophage cells. Rv1954A imparted protection against oxidative and nitrosative stress, thereby enhancing the survival of Ms_Rv1954A inside macrophages. Mice immunized with Ms_Rv1954A showed that splenomegaly and primed splenocytes restimulated with Rv1954A elicited a Th1 response. Infection of Ms_Rv1954A in mice through intratracheal instillation leads to enhanced infiltration of lymphocytes in the lungs without formation of granuloma. While Rv1954A is immunogenic, it did not cause adverse pathology. Purified Rv1954A or Rv1954A knock-in *M. smegmatis* (Ms_Rv1954A) elicited a nearly two-fold higher titer of IgG response in mice, and PTB patients possess a higher IgG titer against Rv1954A, also pointing to its utility as a diagnostic marker for TB. The observed modulation of innate and adaptive immunity renders Rv1954A a vital protein in the pathophysiology of this pathogen.

## Introduction

*Mycobacterium tuberculosis* (*M. tb*), the causative agent of tuberculosis (TB), persists as a latent form in nearly 30% of the global population who may not only serve as reservoir for inadvertent transmission of disease but also develop active TB during immunocompromised conditions. Recent reports have suggested that nearly 10 million new cases of TB are diagnosed annually with almost 1.45 million deaths being reported in 2018 alone (WHO., 2019). There is an exigent need to better understand the pathomechanism of this disease, which advocates an in-depth exploration of the mycobacterial interaction with the host immune system.

Macrophages play a key role in the clearance of bacteria through phagocytosis but paradoxically are the primary targets of *M. tb* infection (Tundup et al., 2008). *M. tb* has evolved mechanisms that enable it to not only avoid phago-lysosomal fusion but also allow the pathogen to remain in a non-replicating state within the macrophages, thereby dodging immunosurveillance (Bussi and Gutierrez, 2019). This is achieved by an arsenal of infectivity factors that modulate the host defense strategies. *M. tb* modulates the activity of the macrophage by dampening the secretion of pro-inflammatory cytokines, which in turn suppress the antibacterial activity of other immune cells. IFN-γ induces the activation of macrophages, thereby leading to phagolysosome formation and generating reactive oxygen species (ROS) that lead to clearance of infection (Winau et al., 2006; Cavalcanti et al., 2012). TNF-α supplements the activity of IFN-γ and generates ROS within the macrophages that exert bacteriostatic effects on pathogens (Delneste et al., 2003; Parameswaran and Patial, 2010). Knockout mice deficient in IFN-γ and TNF-α acquire *M. tb* infections at a higher rate as compared to control mice, pointing to the role of IFN-γ/TNF-α in immunity against TB (Olsen et al., 2016). *M. tb* employs various strategies to dampen this pro-inflammatory cascade to override the host defense system (Hmama et al., 2015). Apart from immune modulation, *M. tb* proteins impair the activity of antigen-presenting cells (APCs) by either suppressing the expression of co-stimulatory molecules or impairing the activity of antigen-presenting molecules (Noss et al., 2000; Hickman et al., 2002).

Studies to explore the immunomodulatory effect of *M. tb* proteins have been at the forefront so as to decipher the role in pathogenesis (Trajkovic, 2004; Hoffmann et al., 2018; Stylianou et al., 2018). Comparative analysis of genome and proteome of non-pathogenic and pathogenic mycobacterial species revealed that *M. tb* evolved through reductive evolution from non-pathogenic mycobacteria (Rahman et al., 2014). Despite the reduction in the genome size, *M. tb* attained pathogenicity by gene co-option whereby several functions were carried out by individual proteins. Additionally, several genes responsible for survival and infectivity expanded in numbers (Saini et al., 2012; Rahman et al., 2014; Singh et al., 2014). In the current study, we examined the function of gene *Rv1954A* that is exclusively present in pathogenic mycobacteria and absent in non-pathogenic mycobacteria. Rv1954A could have a role in *M. tb* infectivity and thus provide insights into the pathomechanism of TB disease. Our study showed the presence of the *Rv1954A* gene in *M. tb* and BCG but absence in non-pathogenic bacteria. The hypothetical protein Rv1954A was expressed in *M. tb* but not in BCG and hence was termed as a “signature protein” of *M. tb*. We elucidated the immunomodulatory role of *M. tb* Rv1954 both *in vitro* and *in vivo*, delineating its role in innate immune modulation and consequent effect on adaptive immune responses.

## Materials and Methods

### Reagents and Other Supplies

Gibco (Thermo Fisher Scientific India Pvt. Ltd., Mumbai, India) supplied all cell culture reagents including DMEM. Merck Limited, Mumbai, India, supplied sarkosyl, imidazole, staurosporine, kanamycin, and isopropyl β-D-1-thiogalactopyranoside (IPTG). BD Biosciences (San Jose, CA, USA) supplied Middlebrook 7H11 agar, Middlebrook 7H9 media, and Middlebrook 7H10 media. ELISA kit, toxicity removal kit, and enzymes were obtained from PeproTech (Rocky Hill, NJ, USA), Norgen (Thorold, ON, Canada), and NEB (Massachusetts, USA), respectively. Plastic wares for cell culture were obtained from Corning (USA). All reagents used were of analytical grade.

### Computational Analyses and Molecular Cloning of Rv1954A

ANCHOR software (https://iupred2a.elte.hu) was used to predict the protein-binding sites in the disordered region, and the IEDB (http://tools.immuneepitope.org/) tool was used to predict T cell/B cell epitopes in the protein of interest. The ORF encoding the *M*. *tb* Rv1954A gene was amplified by polymerase chain reaction (PCR) using forward and reverse primers (Supplementary Table 1). The gene was inserted in *Eco*RI and *Xho*I restriction sites of pET28a to construct a recombinant plasmid pET28a_Rv1954A. The recombinant construct pET28a_Rv1954A was transformed into *E. coli* BL21(DE3) expression strain, and the Rv1954A protein was purified by the Ni-NTA affinity column and eluted with 200 mM imidazole after inducing the culture for 3 h at 37°C with 1 mM IPTG. In order to concentrate the dialyzed protein, 3 kDa cutoff Centricon was used. Contamination of bacterial endotoxin was removed from the concentrated protein by treating with polymyxin B beads followed by estimation of bacterial endotoxin through LAL testing which estimated nil, and the protein was further visualized through SDS-PAGE.

### Generation of *M. smegmatis* Knock-In of *M. tb* Rv1954A

*M. smegmatis* mc^2^155 was obtained from ATCC and maintained as glycerol stocks. These bacterial strains were cultured in Middlebrook 7H9 growth media supplemented with 10% OADC. The pST_Ki_Rv1954A construct was generated using the digested product from the pET28a_Rv1954A construct (Parikh et al., 2013). To make *M. smegmatis* Rv1954A knock-in, electroporation was employed. The positive colonies were selected on Middlebrook 7H11 agar plates containing kanamycin. PCR amplification was used to confirm the positive clones as described previously (Pandey et al., 2017). A three step sequential process was used to confirm the integration of pST_Ki_Rv1954A into the genome of *M. smegmatis*. Firstly, 7H11 agar plates containing kanamycin were used to select the *M. smegmatis*-positive colonies having Rv1954A (Ms_Rv1954A) and vector pST-Ki (Ms_Vc). The positive colonies were then passaged for seven generations on plates containing kanamycin. This was followed by plating and passaging the positive colonies on a kanamycin-negative plate for five generations. Lastly, plating and passaging the colonies for seven generations on a kanamycin plate confirmed the integration of the cassette. Colonies which were confirmed were grown till the log phase and were harvested followed by centrifuging and heating the pellet at 95°C for 30 min and the pellet resuspended in an SDS-PAGE loading dye. Centrifugation of the lysate fraction was done at 13,000 rpm for 10 min, and the supernatant obtained was loaded on 10% tricine gel. Western blotting was used for confirmation of Rv1954A protein by anti-rabbit polyclonal Rv1954A antibody generated in rabbit (described below). Visualization of the blots was done after incubation with anti-rabbit IgG antibody, which were HRP labeled.

### Macrophage Culture and Cytokine Estimation

Murine macrophage cell line RAW264.7 (ATCC) and RAW-ΔTLR4 and RAW-ΔTLR2 were cultured in DMEM supplemented with 10% fetal bovine serum (Gibco), 0.1 mg/mL streptomycin, 10 mM glutamine, and 1× Penta (Gibco) with 5% CO_2_ at 37°C. RAW264.7 cells (5 × 10^4^ cells/well) were cultured with different concentrations of endotoxin-free Rv1954A protein, and supernatant was collected after 24 h for estimation of various cytokines (TNF-α and IL-12). A vial of rRv1954A protein at 10 μg/ml was autoclaved at 121°C, 15 psi of pressure for 30 min to denature/heat inactivate (HI) it, which served as control. rRv1954A protein at 10 μg/ml was treated with Proteinase K (Ambion) at 37°C for 1 h followed by heat inactivation at 95°C for 10 min, which also served as a negative control. 200 ng/ml LPS (*Escherichia coli* O111:B4) obtained from Sigma (USA) was used as a positive control. In another set of experiment, RAW264.7 (ATCC), RAW-ΔTLR4, and RAW-ΔTLR2 (2 × 10^5^) were infected with Ms_Vc or Ms_Rv1954A in a 12-well plate in incomplete DMEM for 4 h at MOI of 1:10 at 37°C followed by three washes with PBS and kept in a complete medium containing gentamicin to kill extracellular bacteria. After 24 h of infection, the supernatant was collected and levels of different cytokines were quantified using murine standard ELISA Development Kit, as per the manufacturer's protocol. Briefly, 96-well ELISA plates were coated with 100 μl of capture antibody and incubated at RT for overnight followed by washing the plates three times with 300 μl PBST (1× PBS pH 7.2, 0.05% Tween 20) and blocking with 1% BSA for 1 h at RT. The plates were then washed thrice with PBST, and addition of 100 μl of previously stimulated supernatant was done in each well incubating at RT for 2 h. Hundred μl of the detection antibody was added after washing five times with PBST followed by adding an enzyme conjugate (100 μl/well), avidin HRP conjugate, for 2 h. The plates were incubated at RT for 2 h. Hundred μl of TMB substrate was added to each well for color development after washing seven times with PBST followed by stopping the reaction with 2N H_2_SO_4_, and absorbance was taken at 450 nm and reference wavelength at 570 nm. The cytokine levels were determined by plotting the curve along with standards.

### Immunofluorescence Staining

RAW264.7, RAW-ΔTLR4, RAW-ΔTLR2, and RAW-ΔTLR2/4 cells (0.5 × 10^6^ cells/well) were seeded on coverslips in a 12-well plate for overnight. In another set of experiment, RAW264.7 which were seeded on the coverslips were incubated with 50 μg/ml or without rat anti-mouse anti-TLR4 antibody for 90 min (Andresen et al., 2016). The cells were treated with 10 μg/ml Rv1954A protein for 6 h followed by washing and fixing the cells with 4% PFA. These fixed cells were blocked with 3% BSA followed by incubating with anti-Rv1954A raised in rabbit (1:500) for 1 h. After washing with PBS, these cells were incubated with Alexa Flour 594-labeled anti-rabbit IgG and DAPI followed by mounting and visualization under a fluorescent microscope.

### Measurement of Reactive Oxygen Species, Nitric Oxide Levels, and Apoptosis

RAW 264.7 cells (2 × 10^5^) infected with Ms_Vc or Ms_Rv1954A in incomplete DMEM for 4 h at MOI of 1:10 at 37°C followed by three washes with PBS and kept in a complete medium containing gentamicin to kill extracellular bacteria. After 12, 24, and 48 h of infection, cells were harvested and washed with PBS. These cells were further processed for determination of levels of ROS or NO generated and apoptosis, as mentioned below. For the determination of the generation of reactive oxygen species (ROS) in RAW264.7 cells, CellROX Orange (Thermo Fisher Scientific India Pvt Ltd, Mumbai, India) was added followed by incubation at 37°C for 30 min. Cells were acquired using a FACSCanto II cytometer (BD Biosciences), and the data were analyzed using FlowJo software (Becton, Dickinson and Company, New Jersey, US). The generation of nitric oxide was determined using Griess reagent as per the manufacturer's protocol. Hundred μl of culture supernatants was added to 100 μl of Griess reagent followed by measuring the absorbance at 570 nm in a spectrophotometer. Apoptosis was measured using Annexin-V-7AAD staining kit (BioLegend, California, USA) as per the manufacturer's protocol. Cells cultured with Ms_Vc or Ms_Rv1954A were harvested, washed with cold PBS, and resuspended in Annexin binding buffer followed by staining with Annexin V-7AAD stain and incubated for 15 min. Treatment of cells with staurosporine (500 nM) served as the positive control. Cells were acquired using FACSCanto II cytometer (BD Biosciences), and the data were analyzed using FlowJo software.

### Extracellular Staining of Surface Markers

RAW 264.7 cells (2 × 10^5^) were infected with Ms_Vc or Ms_Rv1954A in incomplete DMEM for 4 h at MOI of 1:10 at 37°C followed by washing thrice with PBS and kept in complete medium containing gentamicin to kill extracellular bacteria for 48 h. Cells were harvested and treated with fluorescently labeled antibodies against various activation markers. Cells were fixed and analyzed by flow cytometry.

### Mycobacterial Survival Assay

*M*. *smegmatis* mc^2^155 were grown till the log phase and diluted at 1:100 in 7H9 media followed by culturing till OD_600_ reached 0.05 and then were re-inoculated and allowed to grow in culture for 30 h. OD_600_ was taken after every 3 h up to 30 h. RAW 264.7 cells (2 × 10^5^) were infected with Ms_Vc or Ms_Rv1954A in incomplete DMEM for 4 h at MOI of 1:10 at 37°C followed by washing thrice with PBS and kept in complete medium containing gentamicin to kill extracellular bacteria. One ml of 0.025% SDS was used to lyse the cells after 4, 12, 24, and 48 h of infection followed by plating the dilutions on 7H11 agar plates, and colonies were counted and colony-forming units (CFU) were calculated. In another experiment, log phase cultures (OD_600_ of 0.8–1.0) of Ms_Vc or Ms_Rv1954A were diluted 1:100 in 7H9 media and cultured till OD_600_ reached 0.2 and re-inoculated cells were then treated with the indicated concentrations of H_2_O_2_ (5 and 10 mM) or 5 or 10 mM NaNO_2_ for 3, 6, and 9 h, and the cells which survived were grown by plating appropriate dilutions on 7H10 media. The CFU was calculated.

### Mycobacterial Phagocytosis Assay

A 45-min treatment of SYTO-9 (10 μM) (Thermo Scientific) was used to stain the recombinant *M. smegmatis* (100 × 10^6^) Ms_Vc and Ms_Rv1954A. The excess dye was removed by washing the stained cells with PBS, thrice. RAW 264.7 cells (2 × 10^5^) were infected with SYTO-9-stained Ms_Vc or Rv1954A in incomplete DMEM for 4 h at MOI of 1:10 at 37°C followed by washing thrice with PBS and kept in complete medium containing gentamicin in a 12-well plate. Cells were washed three times with PBS, and the internalization of SYTO-9 stained Ms_Vc or Ms_Rv1954A by RAW264.7 cells was analyzed through a flow cytometer. For microscopic visualization of phagocytosis, 10-mm-diameter coverslips were used and RAW 264.7 cells were kept for adherence at 37°C in a CO_2_ incubator. RAW 264.7 were co-cultured with SYTO-9-stained Ms_Vc or Ms_Rv1954A at MOI of 1:10 in incomplete DMEM for 4 h at 37°C. The cells were washed and fixed with 4% PFA in PBS for 30 min, and quenching was done using 50 mM NH4Cl in PBS followed by visualizing the cells through a fluorescence microscope (Nikon Carl Zeiss) (Naqvi et al., 2017).

### Immunizations

All experiments using lab animals were performed according to the guidelines of the Committee for the Purpose of Control and Supervision on Experiments on Animals (CPCSEA), Government of India (CPCSEA guidelines www.envfor.nic.in/divisions/awd/cpcsea_laboratory.pdf), and Institutional Animal Ethics Committee and Institutional Biosafety Committee, National Institute of Pathology, New Delhi, India, approved the protocols (Approval No. NIP/IAEC-1701). All animals used in the experiments were kept in positive-pressure air-conditioned units (25°C, 50% relative humidity, 12-h light/dark cycle). Generation of the polyclonal antibodies against purified recombinant *M. tb*-Rv1954A was done in white New Zealand rabbits by subcutaneous injection of 200 μg/ml of purified recombinant protein emulsified with an equal volume of Freund's incomplete adjuvant followed by two booster immunizations each after 15-days intervals. Two weeks after final immunization, a dot-blot technique was used for quantitative estimation of the antibody titer. For immunization, inbred BALB/c mice (female, 8–12 weeks, 20–25 g) were obtained from the National Institute of Immunology (New Delhi, India). The test group (*n* = 5) was injected subcutaneously with purified recombinant *M. tb*-Rv1954A protein (10 μg/ml) in PBS buffer followed by booster doses (10 μg/ml) after every tenth day till 1 month of primary immunization. The control group (*n* = 5) was sham immunized with PBS only. Use of adjuvant was avoided to minimize the immunomodulatory bias obtained by use of adjuvants (Ciabattini et al., 2016; Knudsen et al., 2016). In another experiment, BALB/c mice (female, 8–12 weeks, 20–25 g) obtained from the National Institute of Biologicals (NIB) Noida, India, were intraperitoneally injected with Ms_Vc (*n* = 6) or Ms_Rv1954A (1 × 10^7^) (*n* = 6) for evaluation of the antigenicity and immunogenicity of Rv1954A protein (Meng et al., 2017; Ruangkiattikul et al., 2017; Dang et al., 2018). After 4 weeks of primary immunization, mice were sacrificed and blood was collected from both sets of mice either immunized with purified recombinant Rv1954A or intraperitoneally injected with Ms_Vc/Ms_Rv1954A, and serum was obtained and stored at −20°C till further use. Another group of mice was also given intratracheal instillation with Ms_Vc or Ms_Rv1954A (1 × 10^6^) followed by a booster dose after 15 days. Mice were sacrificed after 1 month of primary immunization, and lungs were recovered to observe any signs of pathology.

### Isolation of Splenocytes and Estimation of Cytokines

Mice were sacrificed after 30 days of primary immunization, and spleens were recovered. Splenocytes were obtained using standard protocols (Ahmad et al., 2018) for *in vitro* assays. Spleens were recovered and perfused using a 26-gauge needle, and cell suspension obtained using a cell strainer devoid of debris was centrifuged and suspended in RBC lysis buffer (0.84% NH_4_Cl solution). The splenocytes obtained were devoid of erythrocytes and were centrifuged and resuspended in complete media. Splenocytes (1 × 10^6^ cells) were then re-stimulated with recombinant protein (10 μg/ml) for various time points. The supernatants were collected and levels of IFN-γ quantified using the murine standard ELISA Development Kit, as per the manufacturer's protocol described earlier.

In another experiment, splenocytes from mice infected with Ms_Vc or Ms_Rv1954A (0.1 × 10^6^ cells/well) were also seeded in a 96-well plate, stimulated with recombinant protein Rv1954A, and incubated at 37°C for 12, 24, and 48 h. The levels of secreted cytokines TNF-α, IL-6, and IL-12 were quantified using a murine standard ELISA Development Kit (PeproTech, Rocky Hill, NJ, USA) as per the manufacturer's protocol.

### Histological Analysis of Lungs

BALB/c mice (*n* = 6) were given intratracheal infection with PBS, Ms_Vc (1 × 10^6^), or Ms_Rv1954A (1 × 10^6^) in 50 μl PBS. A booster dose of intratracheal infection was also given after 15 days. Mice were sacrificed after 1 month of primary infection. The lungs from the mice were fixed in 4% formalin and were processed for hematoxylin and eosin staining.

### Human Subjects

All the protocols involving the use of samples from human subjects conformed to the Declaration of Helsinki. Approval was granted for all related experiments by the Institutional Ethics Committee (IEC), National Institute of Pathology, New Delhi, India. Informed consent was obtained from all the study participants included in the study. TB patients attending to the DOTS center of All India Institute of Medical Sciences (AIIMS), New Delhi, were enrolled (*n* = 31). Patients attending to other departments with no history of TB or contact with any active TB patient/sample were included as control (*n* = 18). Microscopic examination for presence of acid-fast bacilli in sputum smear abetted with GeneXpert and chest radiography of patients was the primary basis of TB diagnosis. TST status was not known in controls, and considering a high burden setting, 40% of control samples can be safely assumed as latently infected but with no clinical symptom or history of active disease. Blood (5 ml) was withdrawn from the median cubital vein of each participant by a trained phlebotomist. Blood was allowed to coagulate, and serum was isolated and stored at −20°C for further use.

### Estimation of IgG Levels

ELISA was used for estimation of IgG levels in mice and human sera. Briefly, 96-well plates were coated with purified recombinant Rv1954A protein (10 μg/ml) and kept at 4°C overnight followed by washing with PBST (1× PBS pH 7.2, 0.05% Tween 20) three times and blocked with 10% FBS for 1 h at RT again followed by washing. This was followed by washing the plate thrice, and addition of mouse serum samples at 1:100 pre standardized dilution to each well and subsequently incubated for 2 h at RT. After five washes, goat anti-mouse IgG-HRP labeled secondary antibody (Merck Limited, Mumbai, India) at a dilution of 1:10,000 was added and incubated for 1 h. Addition of TMB substrate was done after seven washes followed by stopping the reaction with 2NH_2_SO_4_ and taking absorbance at 450 nm in a spectrophotometer, which correlated with the amount of serum IgG level. In order to assess Rv1954A specificity against sera of TB patients, plates were coated with purified recombinant Rv1954A protein (10 μg/ml) and incubated with patient serum samples at 1:200 dilution. HRP-conjugated secondary antibody at a dilution of 1:10,000 was added and incubated for 2 h. The substrate used was SIGMAFAST™ OPD tablets. Fifty μl of 3 N H_2_SO_4_ was used to stop the reaction. The optical density was measured at a wavelength of 492 nm. To assess the specificity of TB patients' sera against Rv1954A, plates coated overnight with purified recombinant Rv1954A protein (100 μl of 10μg/ml) were incubated with patient and control serum samples at 1:200 dilutions. Appropriate antigen concentration and serum dilution were determined by chequerboard titrations. After 2 h of incubation and 5 subsequent washes, HRP-conjugated secondary antibody at a dilution of 1:10,000 was added and incubated for 1 h. The substrate used was SIGMAFAST™ OPD tablets. Fifty μl of 3 N H_2_SO_4_ was used to stop the reaction. The optical density was measured at a wavelength of 492 nm.

### Statistical Analysis

GraphPad Prism 8 software was used for statistical analysis. ANOVA and Mann–Whitney tests were used to determine statistical significance. A *p* < 0.05 was considered significant, ^*^p < 0.05, ^**^p < 0.01, ^***^p < 0.001, and ^****^p < 0.0001 denote the level of significance.

## Results

### Rv1954A Is Specific to Pathogenic Mycobacteria and Secretory in Nature

The hypothetical Rv1954A protein was examined to understand its role in the modulation of immune response in the host. In order to identify putative proteins for possible coding segments, the open reading frame of the *M. tb* genomic sequence was studied. A comparative study of the *M. tb* Rv1954A putative protein with a database comprising all the known protein sequences showed that it is absent in other species of mycobacteria except BCG. *M. tb* Rv1954A also showed intrinsically disordered regions and encompassed numerous B cell and T cell epitopes (Supplementary Figures S1A,B). The Rv1954A protein is secretory in nature, which was predicted by PredictProtein software (Supplementary Figure S2A). Western blot analysis also showed the secretory nature of Rv1954A as *M. tb* H_37_R_v_ culture filtrate comprised Rv1954A (Supplementary Figure S2B). It was interesting that although BCG had a sequence for Rv1954A, the BCG culture filtrate was devoid of Rv1954A (Supplementary Figure S2B). Cloning of the Rv1954A gene was done followed by expression in the pET28a vector, and the recombinant protein was purified (Supplementary Figure S3). The Rv1954A gene was subcloned in the pST_Ki expression vector followed by electroporation in *M. smegmatis*, and positive constructs having His-tagged Rv1954A (Ms_Rv1954A) or Vector control pST-Ki (Ms_Vc) were cultured (Supplementary Figure S4A).

### Rv1954A Enhances TLR4-Mediated Production of Pro-inflammatory Cytokines in Macrophages

**Figure 1:**
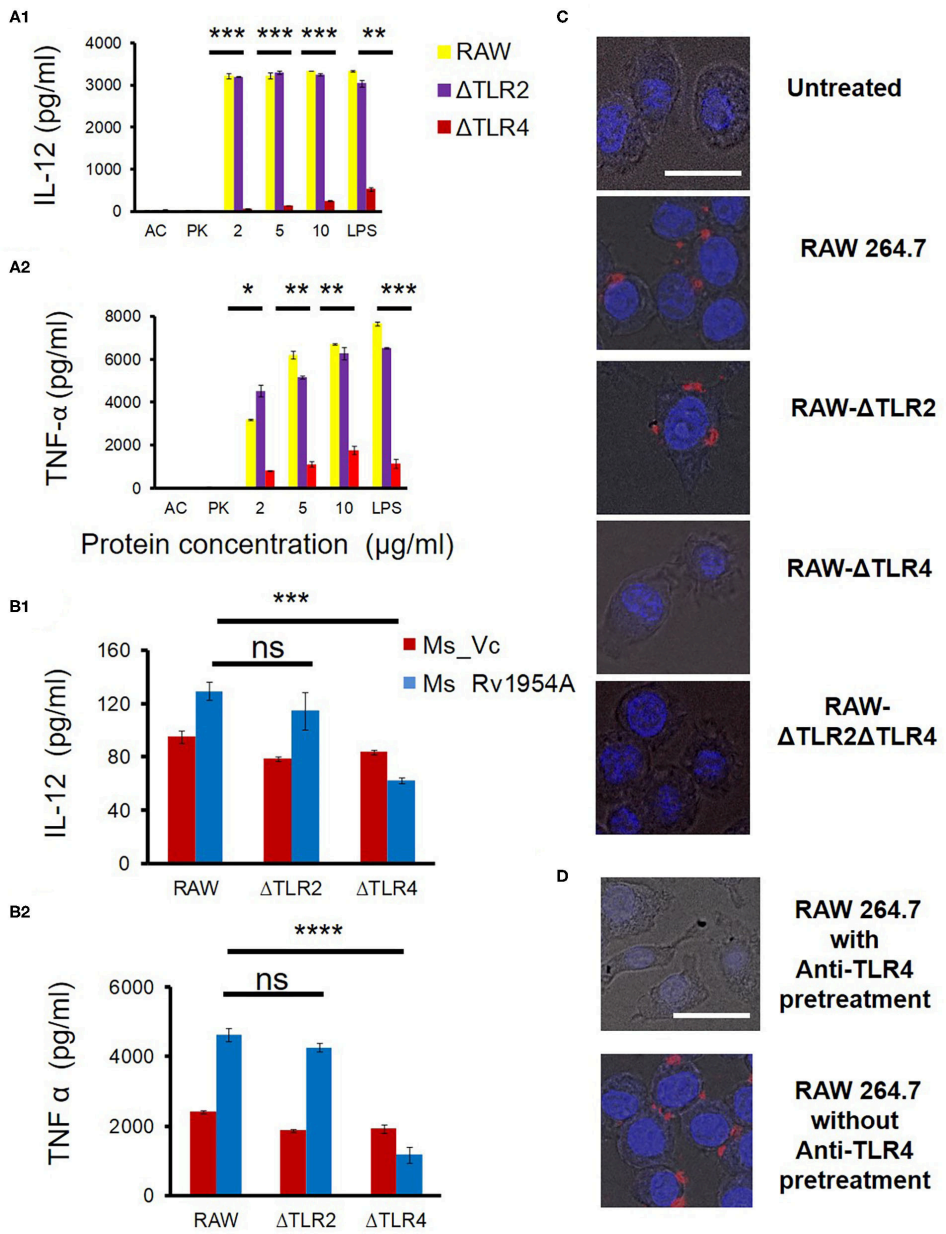
Rv1954A enhances TLR4-mediated production of pro-inflammatory cytokines in macrophages. RAW264.7, RAW-ΔTLR4, and RAW-ΔTLR2 cells were treated with purified Rv1954A protein (2, 5, 10 μg/ml). Autoclaved (AC) protein and proteinase K (PK)-treated protein served as negative controls. LPS treatment served as a positive control. Levels of IL-12 and TNF-α were estimated using ELISA **(A)**. Representative data from three experiments show the concentration of IL-12 and TNF-α as mean ± SEM. Statistical significance was determined with the student *t*-test. **(B)** RAW264.7, RAW-ΔTLR4, and RAW-ΔTLR2 were infected with Ms_Vc and Ms_Rv1954A at an MOI of 1:10. Supernatants were collected after 24 h of infection, and secretion of cytokine levels was estimated through ELISA. Representative data from three experiments show the concentration of IL-12 and TNF-α as mean ± SEM. Statistical significance was determined with the student *t*-test. RAW264.7, RAW-ΔTLR4, RAW-ΔTLR2, and RAW-ΔTLR2ΔTLR4 cells were cultured on coverslips followed by incubation with Rv1954A protein 10 μg/ml for 6 h. The cells were fixed and incubated with anti-Rv1954A followed by staining with Alexa Flour 594 nm and DAPI and visualized by a fluorescent microscope (magnification is 40×, scale bar represents 20 μm) **(C)**. RAW 264.7 cultured on the coverslips were pretreated with or without rat anti-mouse anti-TLR4 for 90 min. Cells were stimulated with 10 μg/ml rRv1954A protein for 6 h followed by fixing and treatment with anti-Rv1954A antibody raised in rabbit tagged with Alexa Flour 594 and DAPI followed by mounting and visualization under a fluorescent microscope (magnification is 40×, scale bar represents 20 μm) **(D)**.

The role of Rv1954A in inducing secretion of cytokine was examined *in vitro* by treatment of RAW264.7 macrophage cells with the rRv1954A protein for 24 h. Even though every batch of protein purified from *E. coli* BL(DE3) was treated with Polymyxin B Agarose for endotoxin removal, any remnant LPS from the *E. coli* cell membrane can lead to induction of cytokine and generate false-positive results. In order to rule this out, a vial of rRv1954A protein at maximum treatment concentrations was autoclaved to denature/heat inactivate (AC) it, which served as control. The rRV1954A protein (10 μg/ml) was treated with Proteinase K (PK) to serve also as a control. The supernatants collected from RAW264.7 cells were estimated for levels of cytokines using ELISA. There was a significant induction of cytokines like IL-12 (Figure 1AI) and TNF-alpha (Figure 1AII) in macrophages, and as expected in negative controls, cytokine levels were at best negligible. In order to identify which TLR is involved in secretion of pro-inflammatory cytokines in macrophages by *M. tb* Rv1954A, RAW264.7 macrophage cells, RAW-ΔTLR4, and RAW-ΔTLR2 were treated with endotoxin-free *M. tb* rRv1954A protein (2, 5, and 10 μg/ml) for 24 h. A significant secretion of IL-12 and TNF-α was observed in RAW264.7 macrophage cells and RAW-ΔTLR2 (Figure 1A). There was a significant reduced secretion of IL-12 and TNF-α in the case of RAW-ΔTLR4 cells.

To independently address whether TLR4 is involved in the induction of cytokine release by *M. tb* rRv1954A, WT and TLR-deficient RAW macrophage cells were infected with Ms_Vc or Ms_Rv1954A. After 24 h of infection, the supernatants were collected, and secretion of cytokines was estimated by ELISA. There was a significant increase in secretion of IL-12 and TNF-α upon infection of RAW264.7 and RAW-ΔTLR2 cells with Ms_Rv1954A compared to Ms_Vc (Figure 1B). However, there was a significant decrease in the secretion of IL-12 and TNF-α upon infection of RAW-ΔTLR4 cells with Ms_Rv1954A compared to Ms_Vc.

Next, RAW264.7 macrophage cells, RAW-ΔTLR2, RAW-ΔTLR4, and RAW-ΔTLR2ΔTLR4 were cultured on coverslips and treated with 10 μg/ml rRv1954A protein for 6 h followed by fixing the cells with 4% PFA. These fixed cells were treated with anti-Rv1954A antibody raised in rabbit tagged with Alexa Flour 594 and DAPI followed by mounting and visualization under a fluorescent microscope, which reveal red puncta on the surface of the cells having DAPI-stained nuclei (blue). These red puncta could be interpreted/indicative as an interacting complex on the surface of the cells. When there was no rRv1954A protein added, such red puncta were not observed (Figure 1C, row 1). Red puncta. Red puncta were observed on the surface of RAW 264.7 cells and RAW-ΔTLR2 incubated with Rv1954A protein (Figure 1C, row 2 and row 3). However, these structures were not observed on the surface of RAW-ΔTLR4 cells and RAW-ΔTLR2 ΔTLR4 cells even in the presence of Rv1954A protein (Figure 1C, row 4 and row 5). As there is presence of red puncta on the surface of RAW 264.7 and RAW-ΔTLR2 cells only and not on TLR4-deficient cells, we concluded that Rv1954A interacts with TLR4 of macrophages. To independently address the formation of these structures, we also pretreated RAW 264.7 cells cultured on the coverslips with or without rat anti-mouse anti-TLR4 for 90 min. Cells were stimulated with 10 μg/ml rRv1954A protein for 6 h, fixed, and treated with anti-Rv1954A antibody raised in rabbit tagged with Alexa Flour 594 and DAPI followed by mounting and visualization under a fluorescent microscope. Pretreatment of anti-TLR4 resulted in no complex formation on the surface of the cells (Figure 1D, row 1) whereas no pretreatment of anti-TLR4 resulted in complex formation (Figure 1D, row 2).

Taken together, our data suggest an interaction of TLR4 with Rv1954A that subsequently leads to secretion of various cytokines.

### Rv1954A Enhances Infectivity of Non-pathogenic Mycobacteria and Leads to Increased Induction of ROS, RNS, and Apoptosis

**Figure 2:**
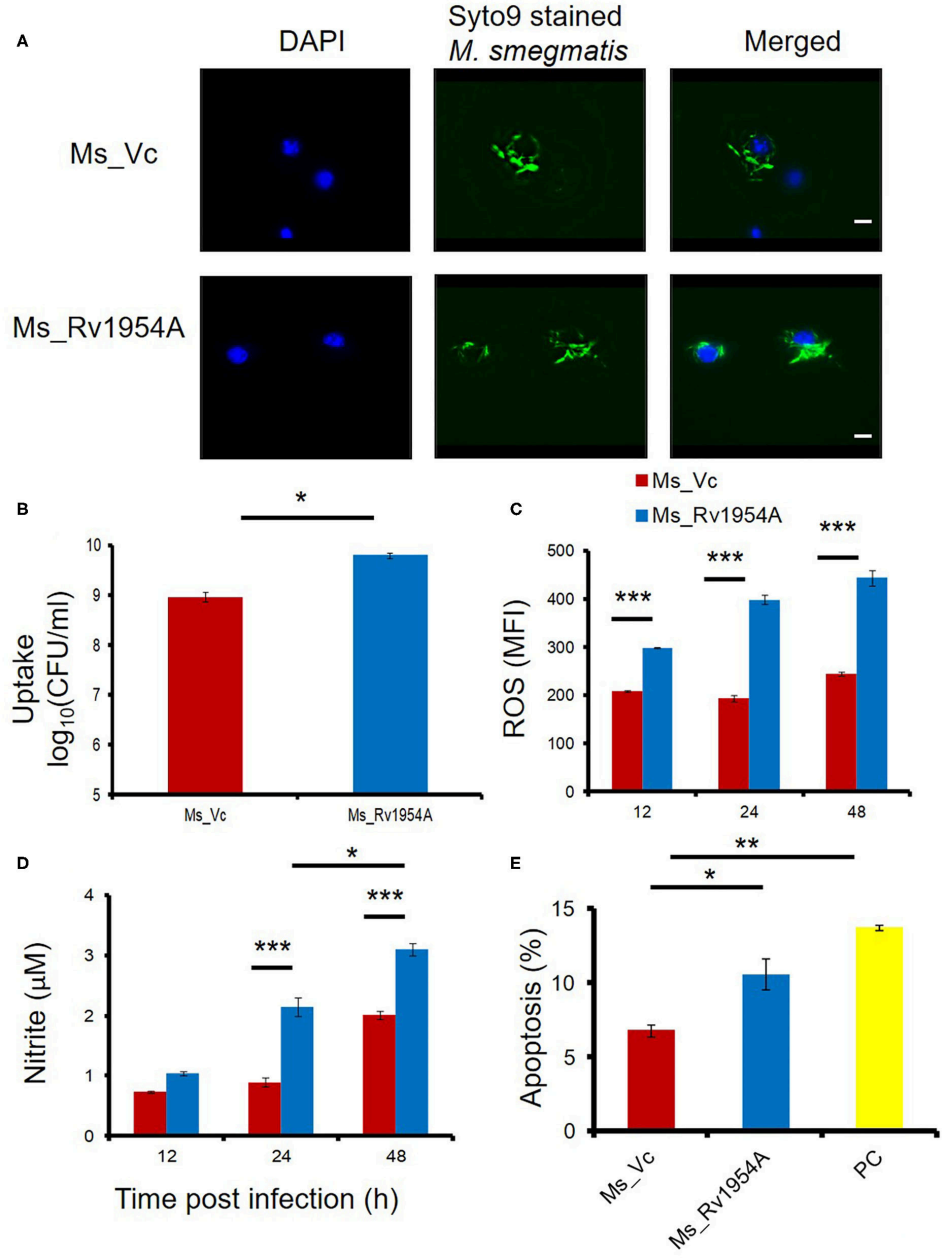
Rv1954A enhances infectivity of non-pathogenic mycobacteria and leads to increased induction of ROS, RNS, and apoptosis. RAW 264.7 cells were infected with fluorescent SYTO 9-stained Ms_Vc and Ms_ Rv1954A. Internalization of Ms_Vc and Ms_Rv1954A by macrophages was visualized through fluorescent microscope (magnification 100×, scale bar represents 5 μm) **(A)** and by CFU assay **(B)**. For estimating the generation of reactive oxygen species, RAW 264.7 cells infected with Ms_Vc and Rv1954A were stained with CellROX Orange. The mean fluorescence intensity (MFI) of ROS generated within the macrophage is represented as mean ± SEM from three separate experiments **(C)**. Two-way ANOVA was used for statistical significance. Levels of nitric oxide (NO) were estimated using Griess reagent assay, after 12, 24, and 48 h post infection of RAW264.7 cells with Ms_Rv1954A or Ms_Vc. The mean fluorescence intensity (MFI) of ROS generated within the macrophage is represented as mean ± SEM from three separate experiments **(D)**. Two-way ANOVA was used for statistical significance. RAW264.7 cells were infected with Ms_Vc or Ms_ Rv1954A at an MOI of 10. After 48 h, cells were stained with Annexin V/7AAD dye and apoptosis was assessed. Data are shown by the percent apoptotic cells as mean ± SEM from three separate experiments. Staurosporine (500 nM)-treated cells were used as a positive control (PC) **(E)**. One-way ANOVA was used for statistical significance. *p* < 0.05 is considered significant, * *p* < 0.05, **p < 0.01. ***p < 0.001.

Growth kinetics was compared for Ms_Vc and Ms_Rv1954A, and there was no difference in doubling time (Supplementary Figure S4B). Ms_Vc and Ms_Rv1954A were fluorescently tagged with SYTO9 and cocultured with RAW264.7 macrophage cells at MOI of 10. SYTO9-stained Ms_Rv1954A were phagocytosed more compared to Ms_Vc, which was confirmed through fluorescence microscopy (Figure 2A). In order to have a quantitative data regarding the infectivity of Ms_Rv1954A, SYTO9-stained Ms_Rv1954A and Ms_Vc were cocultured with RAW264.7 macrophage cells at MOI of 10 for 4 h and harvested followed by running through a flow cytometer. It showed a two-fold higher mean fluorescence intensity (MFI) corresponding to an uptake of SYTO9-stained Ms_Rv1954A as compared to control Ms_Vc (Supplementary Figure S7). The infectivity of Ms_Rv1954A was also assessed by a different approach by infecting RAW264.7 cells with Ms_1954A or Ms_Vc. Macrophages were lysed and plated on agar, and the CFU/ml was examined. The CFU/ml of live bacteria that infected the macrophage cells is shown in Figure 2B. Thus, non-pathogenic *M. smegmatis* attains a comparatively virulent phenotype as visualized by increased infection, which could be attributed to the expression of Rv1954A in recombinant *M. smegmatis*.

There is a generation of reactive oxygen species (ROS) and reactive nitrogen species (RNS) upon infection with mycobacteria in macrophages (Shastri et al., 2018), and apoptosis may take place if it fails to get rid of pathogens (Jamaati et al., 2017). Generation of ROS and NO and apoptosis were assessed after every 12 h after infection with Ms_Rv1954A or Ms_Vc. Infection of RAW264.7 cells with Ms_Rv1954A led to generation of nearly two-fold higher ROS at 24 and 48 h compared to Ms_Vc (Figure 2C). Ms_Rv1954A also induced significantly higher NO at 24 h and 48 h compared to cells infected with Ms_Vc (Figure 2D). Thus, macrophages exhibit stress response by generating ROS and NO after infection with Rv1954A. Viability of macrophages was reduced after infection with Ms_Rv1954A compared to Ms_Vc. The mechanism behind this reduction in viability of RAW264.7 cells was studied by investigating whether cells are undergoing apoptosis, which was assessed by Annexin V/7AAD staining and through flow cytometry. Measuring the percent apoptosis revealed that Ms_Rv1954A induces a significant increase in apoptosis of RAW264.7 cells at 48 h after infection as compared to Ms_Vc (Figure 2E). These results suggest that infection with Ms_1954A leads to stress response in RAW264.7 cells followed by apoptosis.

### Rv1954A Enhances Mycobacterial Survival Inside Macrophages and Accords Protection Against Oxidative and Nitrosative Stress

**Figure 3:**
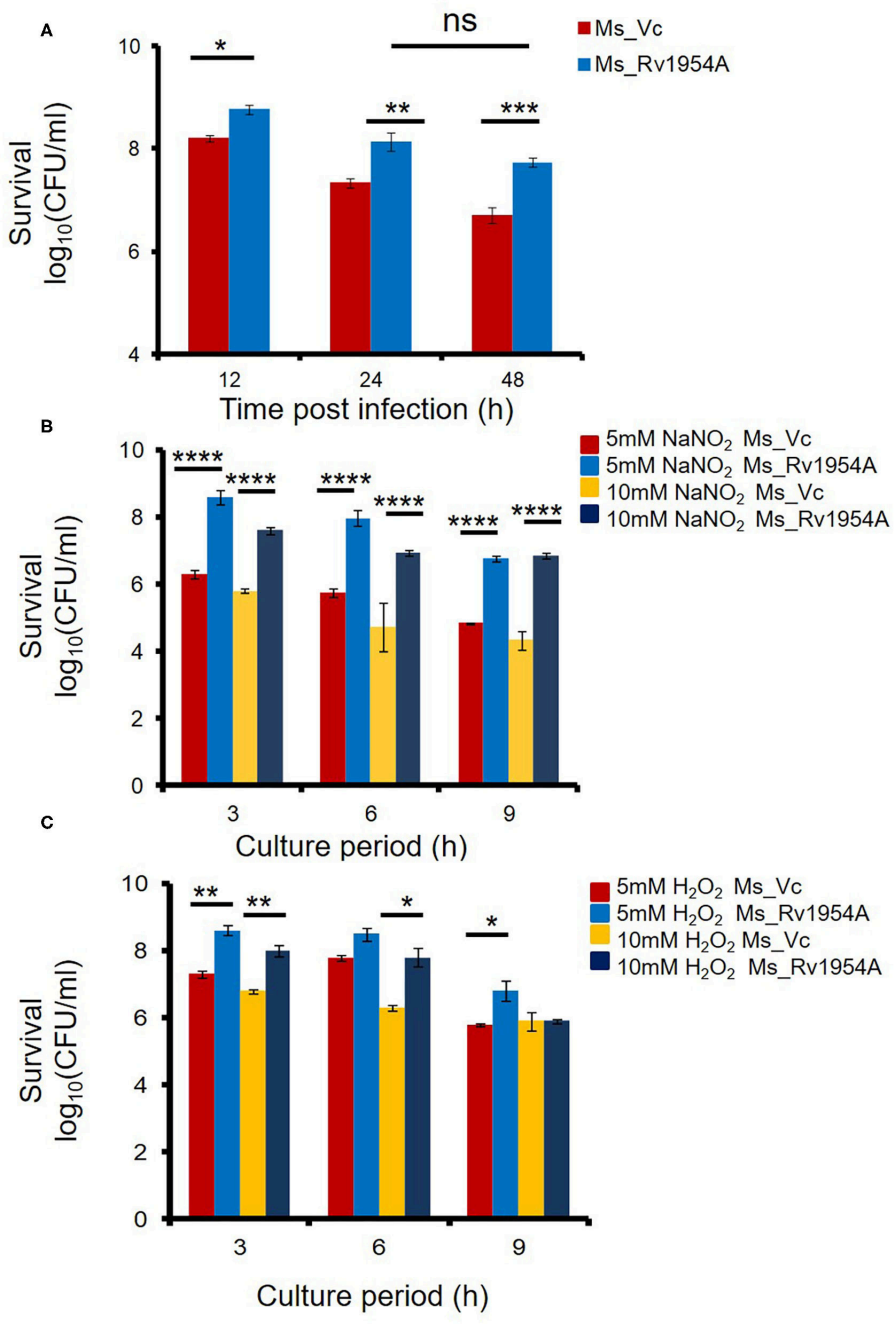
Rv1954A enhances mycobacterial survival inside macrophages and accords protection against oxidative and nitrosative stress. RAW 264.7 cells were infected with Ms_Vc or Ms_Rv1954A, and the colony-forming units/ml were counted after 12, 24, and 48 h. Survival of viable Ms_Vc and Ms_Rv1954A from three experiments is represented as mean ± SEM **(A)**. Ms_Vc and Ms_Rv1954A were given stress by culturing them in the presence of 5 and 10 mM of sodium nitrite. The cell viability was quantitated at 3, 6, and 9 h. Survival in terms of log_10_ CFU/ml as mean ± SEM was represented from three experiments **(B)**. Ms_Vc and Ms_ Rv1954A cells were given oxidative stress by culturing them in the presence of 5 mM and 10 mM of hydrogen peroxide. The cell viability was quantitated at 3, 6, and 9 h. Survival in terms of log_10_ CFU/ml was represented from three experiments as mean ± SEM **(C)**. Two-way ANOVA was used for statistical significance. *p* < 0.05 is considered significant, *p < 0.05, **p < 0.01. ***p < 0.001 and ****p < 0.0001.

Experiments were designed to evaluate the role of Rv1954A in survival under stress. As can be seen, the survival of Ms_Rv1954A was significantly enhanced at 24 h and 48 h compared to Ms_Vc (Figure 3A). Colony formation (CFU/ml) was used to enumerate survival of Ms_Rv1954A within macrophages at different time points after infection. CFU obtained represented the number of bacteria that survived within the macrophages. The observed data suggest that expression of Rv1954A imparts infectivity character to non-pathogenic *M. smegmatis* that translates into increased survivability within the macrophage.

An increased amount of ROS and RNS in macrophages can successfully eliminate *M. smegmatis* infection (Jordao et al., 2008). However, we observed that Ms_Rv1954A survives for longer compared to Ms_Vc within the macrophages (Figure 3A) despite enhanced ROS and RNS levels. This observation was revalidated by *in vitro* generation of RNS- and ROS-mediated stress. Treatment of Ms_Rv1954A or Ms_Vc with sodium nitrite (5 and 10 mM) and hydrogen peroxide (5 and 10 mM) for up to 9 h imitates the induction of RNS and ROS stress, respectively. The viability of Ms_Vc or Ms_1954A in NaNO_2_ and H_2_O_2_ was measured through CFU. There is an increase in CFU of Ms_Rv1954A as compared to Ms_Vc after treatment of the cultures of Ms_Rv1954A and Ms_Vc with 5 mM NaNO_2_ or with 10 mM NaNO_2_ for 3, 6, and 9 h (Figure 3B). Treatment of cultures with H_2_O_2_ (5, 10 mM) also resulted in significantly higher CFU of Ms_Rv1954A cells as compared to Ms_Vc cells (Figure 3C). Thus, Ms_Rv1954A is more resistant to RNS or ROS stress compared to Ms_Vc, which suggests that Rv1954A accords with protection against oxidative and nitrosative stress within macrophages. Thus, Rv1954A proteins likely help mycobacteria survive under stress conditions created within immune cells after infection.

### Upregulation of Molecules Associated With Cell Maturation, Co-stimulation, and Antigen Presentation by Rv1954A in Macrophages

**Figure 4:**
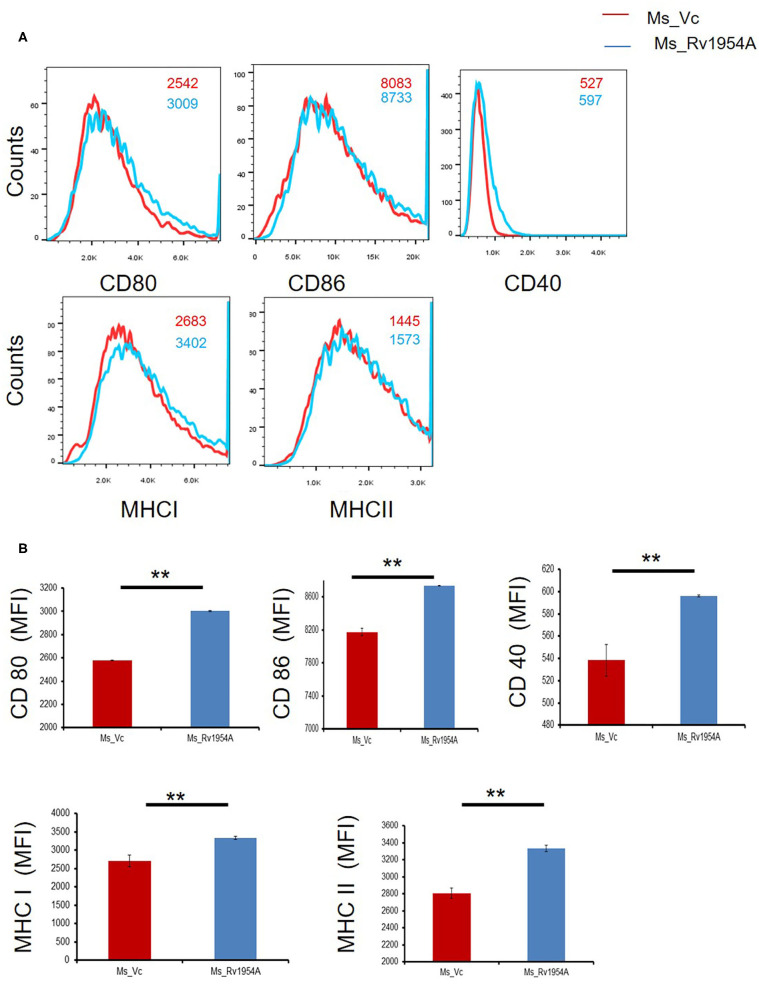
Upregulation of molecules associated with cell maturation, co-stimulation, and antigen presentation by Rv1954A in macrophages. RAW264.7 cells were infected with Ms_Vc and Ms_Rv1954A at an MOI of 10:1. Cells were harvested after 24 h of infection. The frequency of CD40^+^, CD80^+^, CD86^+^, MHCI, and MHCII markers was determined through a flow cytometer. Representative facs plots show the mean fluorescence intensity of cellular markers **(A)**. Representative data show the mean fluorescence intensity of cellular markers as mean ± SEM **(B)**. Statistical significance was determined with one tailed Mann–Whitney test. A *p* < 0.05 was considered significant. **p < 0.01.

In order to study the role of Ms_Rv1954A on activation of macrophages, flow cytometry was employed. CD80, CD86, and CD40 which are co-stimulatory markers along with MHC I and MHC II, which are antigen-presenting molecules, were examined. Mean fluorescence intensity signifying the estimate of surface expression of CD80, CD86, CD40, MHC I, and MHC II on macrophages increased significantly upon infection with Ms_Rv1954A as compared to Ms_Vc (Figure 4). It clearly depicts that Rv1954A play an important role in activation of macrophages by enhancing the expression of CD80 and CD86. There was an upregulation of CD40 by Rv1954A, which signifies that Rv1954A likely enhances the association of APCs with T cells during antigen presentation. There was also an increase in MHC I and MHC II by Rv1954A, pointing to their role in modulation during antigen presentation to CD4^+^ and CD8^+^ cells.

### Rv1954A Generates Th1-Biased Immune Response in Mice

**Figure 5:**
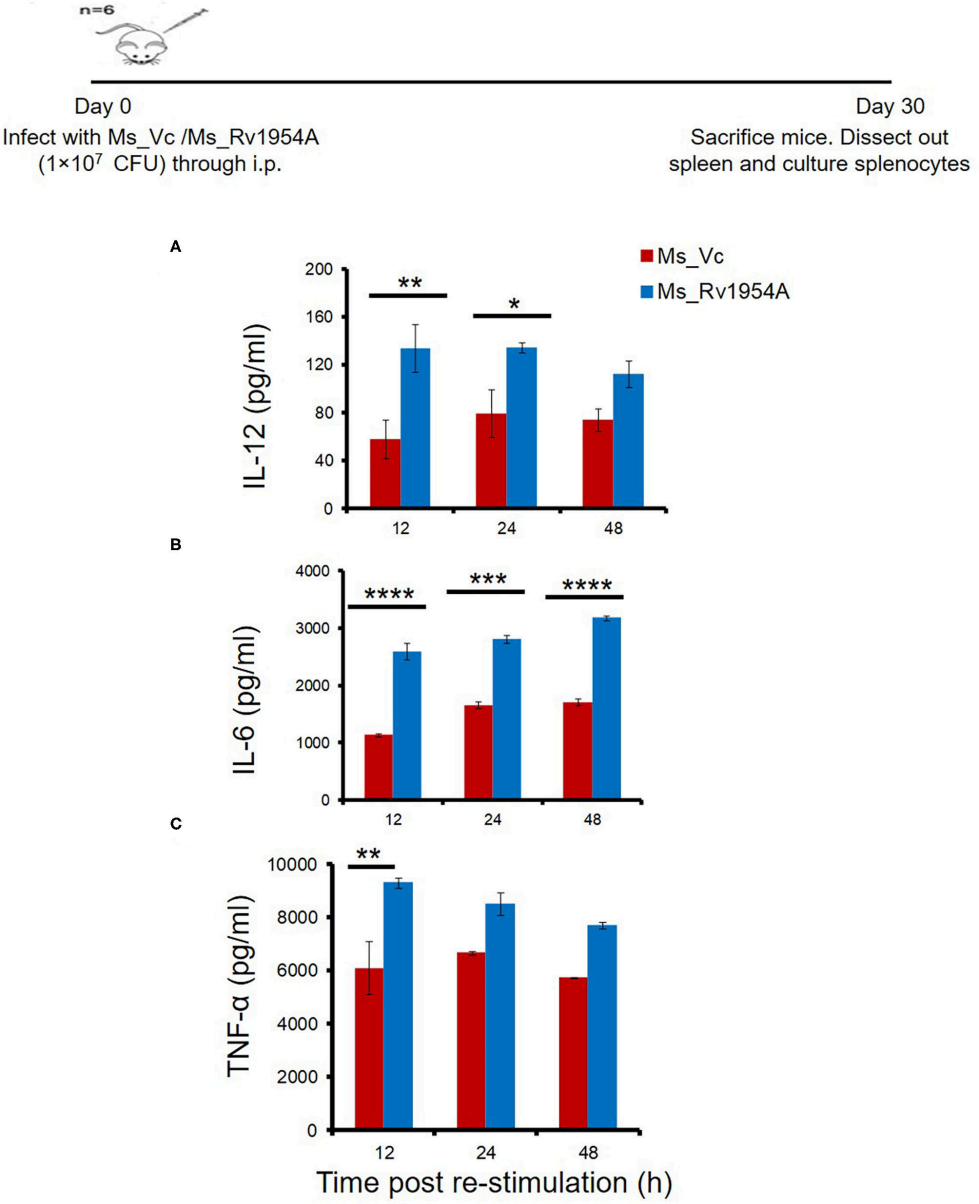
Rv1954A generates Th1-biased immune response in mice. Spleen were recovered from mice (*n* = 7) infected with Ms_Vc (1 × 10^7^) and Ms_Rv1954A (1 × 10^7^). Primed splenocytes were *in vitro* stimulated with Rv1954A protein for 12, 24, and 48 h. Secretion of IL-12 **(A)**, IL-6 **(B)**, and TNF-α **(C)** was estimated through ELISA. The concentrations of IL-12, IL-6, and TNF-α are represented as mean ± SEM from three experiments. Two-way ANOVA was used for determination of statistical significance. *P* < 0.05 is considered significant, *p < 0.05, **p < 0.01, ***p < 0.001, and ****p < 0.0001, and nd means not detected.

In order to study the immunomodulatory role of Rv1954A in the generation of lymphocyte subpopulations, mice were immunized either with rRv1954A protein or with recombinant *M. smegmatis* (Ms_1954A). Administration of Ms_Rv1954A resulted in splenomegaly (Supplementary Figure S5A) in mice observed at necroscopy (1 month after infection). There was also an increase in the number of splenocytes (Supplementary Figure S5B) as compared to Ms_Vc. The primed splenocytes were further re-stimulated with Rv1954A proteins (5 μg/ml) for 12, 24, and 48 h which induced significantly higher IL-12 (Figure 5A), IL-6 (Figure 5B), and TNF-α (Figure 5C). Thus, Rv1954A mounts a strong pro-host Th1 response. Moreover, estimation of IFN-γ from culture supernatants also revealed an enhanced secretion from Rv1954A-immunized animals (Supplementary Figure S6).

### Infection of Ms_Rv1954A Through Intratracheal Instillation in Mice Leads to Enhanced Infiltration of Lymphocytes in the Lungs

**Figure 6:**
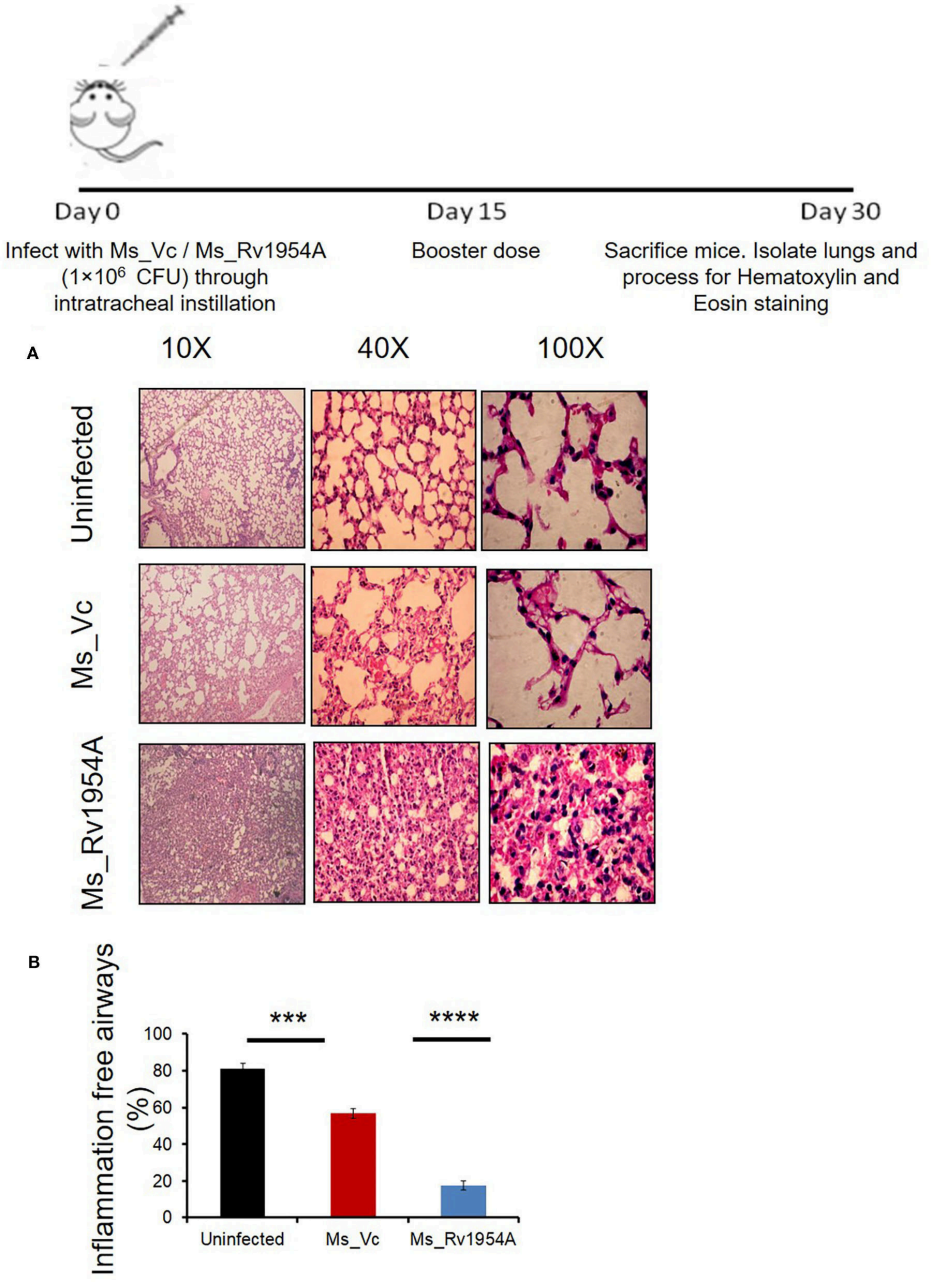
Infection of Ms_Rv1954A through intratracheal instillation in mice leads to enhanced infiltration of lymphocytes in the lungs. BALB/c mice (*n* = 6) that were injected with either phosphate-buffered saline (uninfected) or Ms_Vc (1 **×** 10^7^) or Ms_Rv1954A cells (1 × 10^7^) intratracheally. Lungs were recovered and washed in PBS followed by fixing in 10% formalin solution. Paraffin-embedded lungs were fine sectioned followed by staining with hematoxylin and eosin (HE) solution. The images were captured for at least 5 different fields at different magnifications **(A*)***. Quantitative representation of the inflammation-free airways in the lungs of mice after infection with Ms_Vc or Ms_Vc intratracheally through hematoxylin and eosin staining **(B)**. Data are represented as mean ± SEM. Statistical significance was determined with student's *t*-test, ***p < 0.001 and ****p < 0.0001.

After observing a good immunological response of Rv1954A and enhanced survival of Ms_Rv1954A in macrophages, we checked its effect by aerogenic route in order to mimic actual infection. Comparison of histological analysis of the lungs of mice infected with Ms_Rv1954A and Ms_Vc through intra-tracheal instillation showed enhanced infiltration of lymphocytes into the lung parenchyma in the case of mice infected with Ms_Rv1954A (Figure 6A). Granuloma-like structures were not seen in any of the hematoxylin and eosin-stained samples of the lung tissues. The inflammatory airways in lungs of mice infected with Ms_Rv1954A were visualized by a blinded observer and were found significantly less in comparison to mice infected with Ms_Vc (Figure 6B). These observations clearly suggest that Ms_Rv1954A is more immunogenic compared to Ms_Vc but at the same time is not so virulent that it can cause any adverse pathology.

### Rv1954A Induces IgG Response and Sero-Specificity Determines Its Possible Diagnostic Candidature

**Figure 7:**
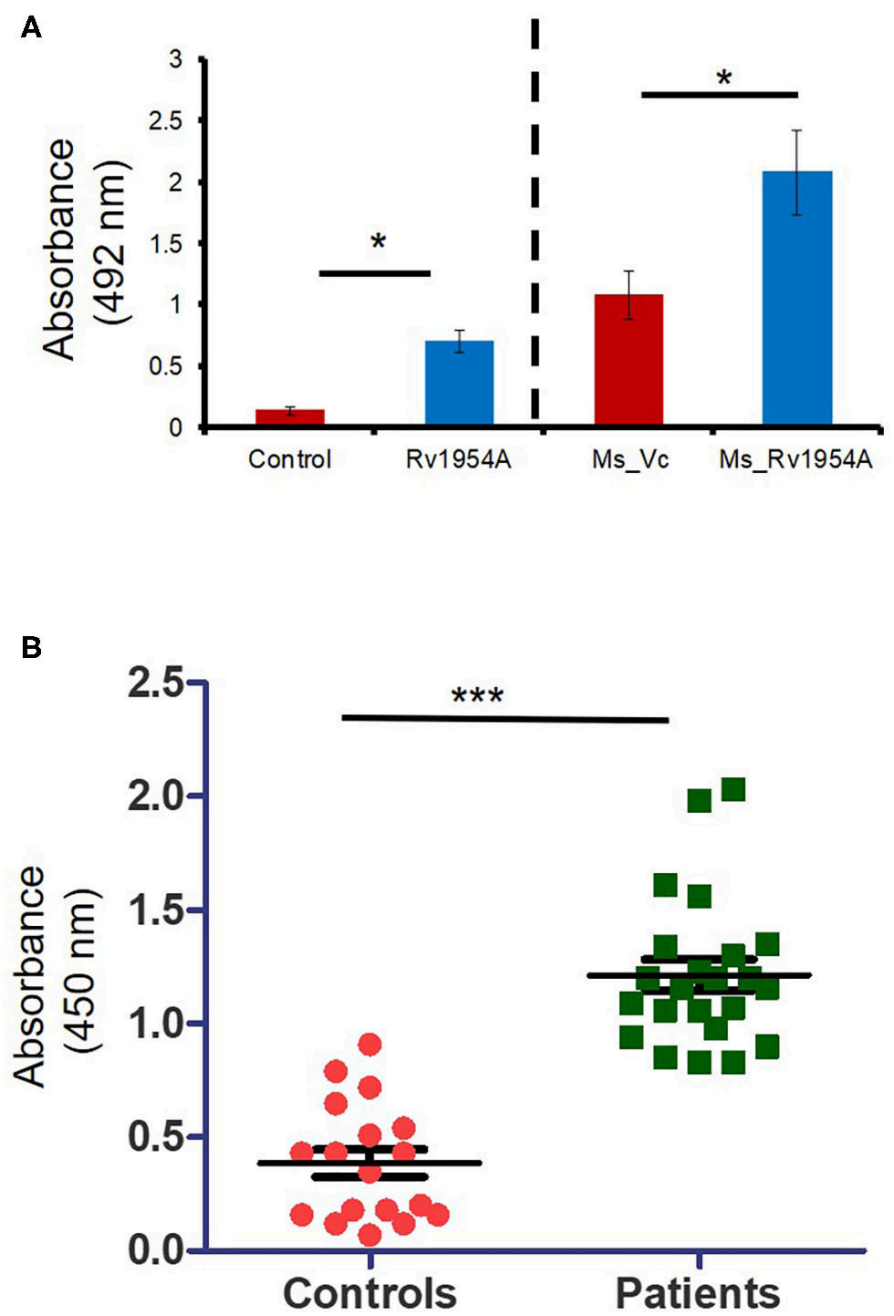
Induction of IgG response exhibiting sero-specificity by Rv1954A. *M. tb* Rv1954A protein was immunized in BALB/c mice or mice infected with Ms_Rv1954A and Ms_Vc. The mouse sera having IgG was tested using ELISA **(A)**. Sera were obtained from pulmonary TB patients (*n* = 31) or control volunteers (*n* = 19). Immunoreactivity of human sera against Rv1954A was tested using ELISA **(B)**. Data are represented as mean ± SEM. One-tailed Mann–Whitney test was used for statistical significance. *p* < 0.05 is considered significant, *p < 0.05, ***p < 0.001.

Having observed the role of Rv1954A in cell-mediated immunity, we explored the role of Rv1954A in humoral immune response. ELISA was performed to measure the immunoglobulin G (IgG) level in the sera of mice immunized with either purified Rv1954A protein or Ms_Rv1954. Mice immunized with Rv1954A protein showed a nearly one-fold higher titer of IgG in the sera as compared to control mice. Mice immunized with Ms_Rv1954A showed a nearly two-fold higher titer of IgG as compared to mice injected with Ms_Vc cells (Figure 7A). Ability of Rv1954A to elicit an IgG response led us to validate whether Rv1954A-specific IgGs could be employed as a diagnostic biomarker. A comparative humoral immune response against the Rv1954A protein in pulmonary tuberculosis (PTB) patients and control population revealed a significantly higher IgG titer in sera of all the PTB-positive patients compared to sera of the controls (Figure 7B). These results point to the likely utility of Rv1954A as a novel diagnostic marker for TB.

## Discussion

In order to eradicate TB by 2035, as envisaged under the Sustainable Development Goals of UNO and the WHO End TB Strategy, major focus of research includes areas such as discovering new drugs, repurposing drugs, discovering new vaccines, and administering vaccines through different routes to enhance its efficacy (Khan et al., 2019; Kumar et al., 2019; Darrah et al., 2020; Sheikh et al., 2020). Our earlier intensive comparative computational analysis revealed proteins that are specific to *M. tb* pathogen and absent in all other mycobacterial species (Rahman et al., 2014). Pathogen specificity puts these proteins at the forefront of process of pathogenesis demanding exploration in detail. In this study, we evaluated the immunomodulatory role of *M. tb* antigenic protein Rv1954A, which is present but not expressed in the BCG strain. Rv1954A is highly antigenic, which is revealed through *in silico* analysis for epitope search and antigenicity. Therefore, these pathogenic proteins need to be researched in detail for their possibility to be used in diagnostics and as vaccine candidates.

Our study revealed that treatment of macrophages with Rv1954A led to release of pro-inflammatory cytokines like IL-12, IL-6, and TNFα in a dose- and time-dependent manner by interacting *via* TLR4. The same results were corroborated in mice infected with non-pathogenic *M. smegmatis* expressing Rv1954A (Ms_Rv1954A) wherein sensitized lymphocytes also evoked a pro-inflammatory response after *in vitro* treatment with antigens.

*M. tb* Rv1954A also activates innate immunity by activating macrophages, which is evident by increased expression of co-stimulatory markers such as CD80, CD86, and CD40. This suggests that treatment of Rv1954A activates macrophages, and this protein is exploited by these host sentinels to produce an efficient microbicidal response. The initial innate immune modulatory response by Rv1954A in macrophages is likely translated later into efficient adaptive response by lymphocytes, which determines the outcome of the interaction. Moreover, the immune response usually gets skewed toward either Th1 or Th2, depending on their physiochemical properties/adjuvant used (Freeman et al., 1993; Levine et al., 1995). We deliberately omitted the use of any adjuvant as mycobacterial proteins have self-adjuvanting properties that we have observed earlier and have also been previously reported (Lee et al., 2014). Induction of hallmark pro-inflammatory cytokine IFN-γ in immunized mice was also observed, which clearly depicts pro-host immune modulatory effects of the antigenic protein (Supplementary Figure S7). TNFα, along with INF-γ, produces NOS, which enhances a bacteriostatic effect of macrophages. TNF-α also has a role in modulation of migratory potential leading to granuloma formation, which limits the bacteria from further transmission (Cavalcanti et al., 2012). This cytokine also regulates other inflammatory cytokines like IL-1 and IL-6 during *M. tb* infection. Despite all pro-host effects, this cytokine needs strict regulation as excessive levels can lead to immunopathology with worsening of clinical symptoms. IL-12 is required for the expansion and optimal activation of Th1 response by inducing the production of IFNγ. Apart from this, IL-12 also facilitates dendritic cell migration along with chemokine production to control mycobacterial infections. IL-6 production is also critical during early *M. tb* infection as it affects protection by modulating cytotoxic T cell differentiation. IL-6 exhibits a protective role in the early phase while causing pathology in the chronic phase of TB infection, thereby stating it to be controversial (Zhang et al., 1994). Though IL-17 has emerged as another critical cytokine that imparts mucosal protection against *M. tb*, the effect of Rv1954A on that subset is yet to be validated. The production of these pro-inflammatory cytokines suggests a switch toward the Th1 type of response that is critical for protection against intracellular pathogens like *M. tb*.

Our results suggested that Ms_Rv1954A survives for a prolonged duration within the macrophage, indicating an increase in infectivity. These infected macrophages also reveal an enhanced bactericidal response, which was paradoxical to enhanced growth of recombinant bacteria inside macrophages. Thus, generation of reactive oxygen species (ROS) and reactive nitrogen species (RNS) takes place in the macrophage to get rid of the pathogen (Shastri et al., 2018) or may become apoptotic if it does not eliminate the pathogen (Jamaati et al., 2017) upon infection with *M. tb*. This suggested that Rv1954A somehow imparts resistance to these oxidative and nitrosative stresses, which was validated by inducing stress to the recombinant bacteria Ms_Rv1954A. The mechanistic details to deciphering the role of Rv1954A in conferring resistance to stress are further warranted. These results also provide a mechanism of apoptosis induction in macrophages upon infection with recombinant Ms_Rv1954A. Enhanced infectivity acquired by Ms_Rv1954A probably leads to activation of apoptosis. Apoptosis acts as a host defense mechanism as apoptosis controls bacterial replication. Apoptotic macrophages are an important source of bacterial antigens, which stimulate *M. tb*-specific T-cell immunity (Winau et al., 2006). These attributes indicate that vaccine candidates expressing Rv1954 could survive longer and eventually generate efficient innate and adaptive immune responses. One of the possible setbacks could be infectivity-mediated pathology. Interestingly, upon aerosol infection with Ms_Rv1954A, we observed enhanced infiltration owing to enhanced immunogenicity, but no overt pathology mediated by knock-in of Rv1954A.

All evidence emerging was suggestive of a Th1 type of response that advocates use of Rv1954 as a probable vaccine candidate. The observed immunological characteristics strongly suggest a novel role for this signature protein Rv1954A. In addition to cell-mediated immunity, a humoral branch of the immune system is also activated by Rv1954A. There are recent reports which suggest that antibodies can have a positive effect on the immune responses against *M. tb*. B cells are multifaceted because of their ability to present antigen, secrete antibody, and cytokine, exerting a significant effect on T cell-mediated immunity, which is considered critical for TB control (Igietseme et al., 2004; Lund and Randall, 2010; Chan et al., 2014). Taking these findings into consideration, we expected antibodies against Rv1954A in sera of TB patients. We found a clear non-overlap in IgG reactivity in PTB patients and controls, which clearly reflects that response by Rv1954A is highly specific. The sero-specificity of Rv1954A suggested that it can be used as a diagnostic marker for TB like resistin which we have earlier reported (Ehtesham et al., 2011). Considering the variable efficacy of BCG, a signature protein of *M. tb* such as Rv1954A can be genetically engineered into BCG, which may solve some of the drawbacks of BCG.

**Figure 8:**
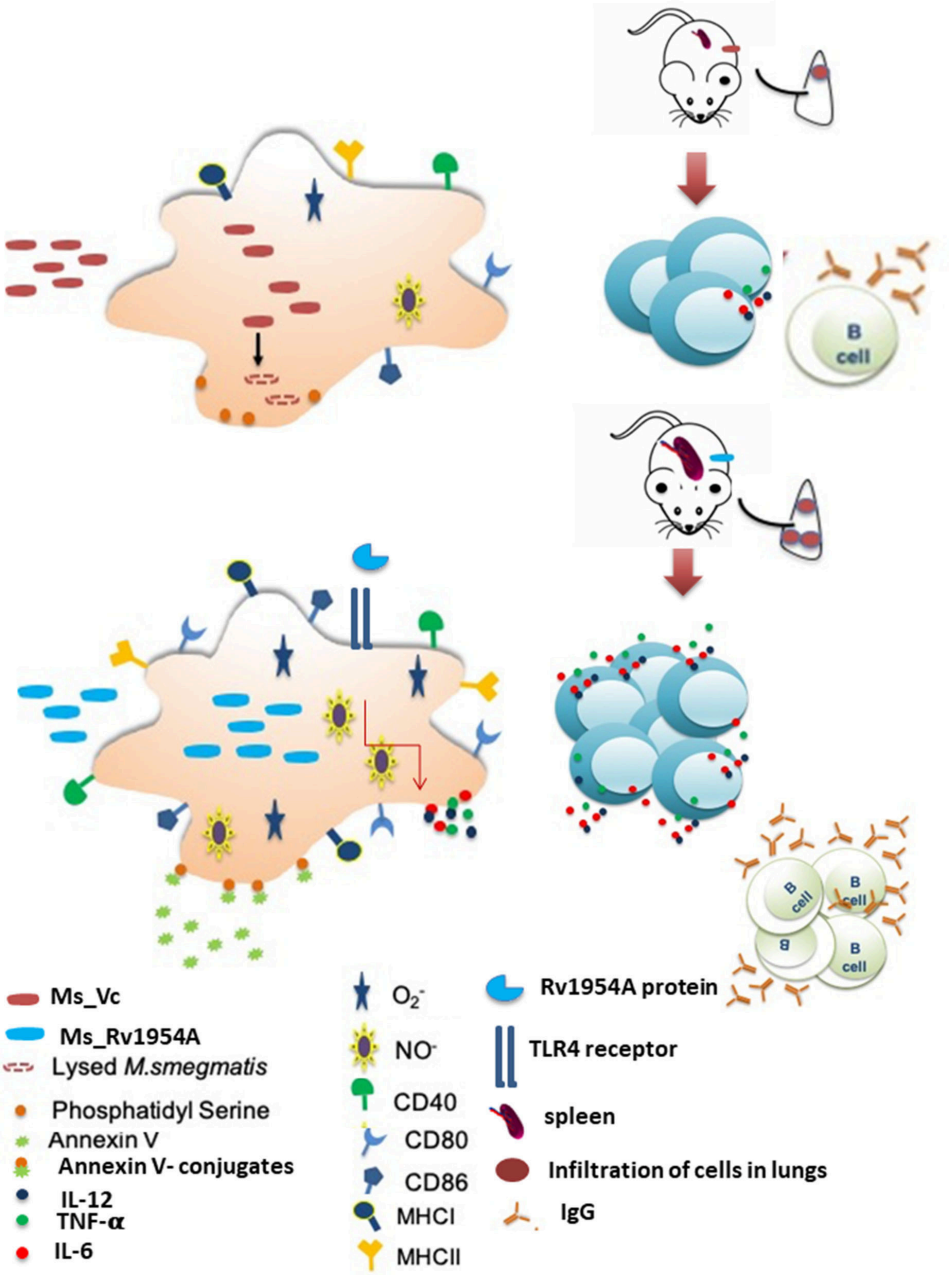
Model depicting the role of Rv1954A in modulating immune responses. Our studies showed that *M. tb* Rv1954A protein is a secretory protein that elicits high levels of Th1 cytokines in macrophages through the TLR4-mediated pathway. Rv1954A knock-in *M. smegmatis* (Ms_Rv1954A) was taken up more by macrophages and endured stress response but showed higher survival in macrophages. Ms_Rv1954A induced increased expression of early activation marker CD69, co-stimulatory molecule CD40, antigen-processing molecule (MHCII), and CD80/CD86 molecules associated with modulation of T cell activity in macrophages. Intratracheal instillation of Ms_Rv1954A also resulted in infiltration of cells in the lungs without any granuloma formation. *M. tb* Rv1954A showed specificity to the sera obtained from TB patients.

Thus, we can conclude that *M. tb* Rv1954A has an immunomodulatory role (Figure 8). It can induce pro-host immune response under both *in vitro* and *in vivo* conditions. However, we have not addressed the role Rv1954A in the context of *M. tb* infection. Moreover, the emerging and substantial role of “trained immunity” in TB control and vaccine developments has drawn impetus again on innate immunity that was overshadowed by adaptive immunity (Koeken et al., 2019; Netea et al., 2020). Exploring epigenetic and metabolic programming of innate immune cells by mycobacterial effector proteins will elucidate the molecular mechanism to generate efficacious vaccines for better control of tuberculosis (Lerm and Netea, 2016; Covian et al., 2019).

## Data Availability Statement

All datasets generated for this study are included in the article/Supplementary Material.

## Ethics Statement

The studies involving human participants were reviewed and approved by National Institute of Pathology Institutional Ethics Committee. The patients/participants provided their written informed consent to participate in this study. The animal study was reviewed and approved by National Institute of Pathology Institutional Animal Ethics committee.

## Author Contributions

NE, SR, and SH conceptualized and designed the research. SA, NN, BA, JA, and AA performed the experiments. SA, NN, AA, PK, and JS carried out the data analysis. SA, NN, AA, JS, SH, DM, and NE wrote the manuscript. All authors contributed to the article and approved the submitted version.

## Conflict of Interest

SR was employed by BioInception Pvt. Ltd., UK. The remaining authors declare that the research was conducted in the absence of any commercial or financial relationships that could be construed as a potential conflict of interest.

## Supplementary Material

Supplementary Figure S1 *M.tb* Rv1954A revealed its antigenic potential through *in silico* analysis. **(A)** B-cell and T cell epitope prediction in Rv1954A by IEDB tool (http://tools.immuneepitope.org). **(B)** Anchor server was used to predict presence of protein binding sites in disordered regions (https://iupred2a.elte.hu).

Supplementary Figure S2 Secretory nature of Rv1954A is revealed through *in silico* and western blot analysis. **(A)** Localization prediction of Rv1954AA by PredictProtein tool (https://www.predictprotein.org/) depicting its secretory nature. **(B)** Western blot image depicting the expression of Rv1954A in CFP of *M. tb* H_37_Rv. A distinct band of 10 KDa is observed in CFP of *M. tb* H_37_Rv but not in the CFP of BCG. WCL, Whole Cell Lysate; CFP, Culture Filtrate Protein; M, marker.

Supplementary Figure S3 Construction and expression of Rv1954A knock-in *M. smegmatis*. **(A)** The elution of recombinant *M.tb* Rv1954A depicted by SDS PAGE. A distinct band is observed at 10 KDa (M: marker; FT: flow through; W1: Wash1; W2: Wash 2; W3: Wash 3; W4: Wash 4 E1-E3: Elutions). **(B)** Confirmation of recombinant *M.tb Rv*1954A using western blot. **(C)** Molecular characterization of pST_Ki_Rv1954A knock-in *M. smegmatis*. Confirmation of Subcloning of Rv1954A in pST-Ki expression vector using colony PCR (PC: positive control; L: DNA ladder; 1-4: colony). **(D)** Confirmation of pST-Ki_ Rv1954A in knock-in *M. smegmatis* using colony PCR. PC, positive control; L, DNA ladder; 1-4: Colonies 1-4.

Supplementary Figure S4 Rv1954A of knock-in *M. smegmatis* Ms_Rv1954A expresses Rv1954A. **(A)** Western blot confirmation of *M.tb* 1954A using in-house specific polyclonal antibody raised in rabbit. Note the presence of a band corresponding to 10 KDa in Ms_Rv1954A lane. **(B)** Growth curve of Ms_Rv1954A as compared to vector control Ms_Vc. Statistical significance was determined with two-way ANOVA. Note the absence of any significant difference in terms of growth kinetics between Ms_Rv1954A and Ms_Vc.

Supplementary Figure S5 Splenomegaly and increased number of splenocytes is caused by Ms_Rv1954A. BALB/c mice (*n* = 6) that were injected with either PBS (uninfected) or Ms_Vc (1 × 10^7^) or Ms_Rv1954A (1 × 10^7^). Spleen was recovered after 1 month. **(A)** Representative picture depicting splenomegaly in the mice infected with Ms_Rv1954A as compared to Ms_Vc. **(B)** The number of splenocytes was counted after making single cell suspension using trypan blue exclusion method. Representative data show the number of splenocytes as mean ± SEM. Statistical significance was determined with one tailed Mann-Whitney test. *p* < 0.05 was considered significant, ^**^*p* < 0.01.

Supplementary Figure S6 Induction of secretion of IFN-γ from re-stimulated splenocytes by Rv1954A. BALB/c mice (*n* = 5) were immunized with purified recombinant Rv1954A proteins (10 μg/ml). Splenocytes (1 × 10^6^) isolated from mice were cultured in absence or presence of Rv1954A protein (2, 5, 10 μg/ml) for 48 h and the levels of IFN-γ were estimated by ELISA. Representative data show IFN-γ secretion, as mean ± SEM. Statistical significance was determined with one tailed Mann-Whitney test. *p* < 0.05 was considered significant, ^*^*p* < 0.05 and ^**^*p* < 0.01.

Supplementary Figure S7 Increased survival of Ms_Rv1954A in infected macrophages. RAW264.7 cells were co-cultured with SYTO-9 stained Ms_Rv1954A or Ms_Vc at MOI of 10:1. The uptake of Ms_Vc and Ms_Rv1954A cells within RAW264.7 macrophage cells was assessed by flowcytometer after 12, 24, and 48 h. Representative data from three experiments show mean fluorescent intensity (MFI) of fluorescently tagged viable Ms_Vc and Ms_Rv1954A as mean± SEM. Statistical significance was determined with two-way ANOVA. *p* < 0.05 was considered significant, ^***^*p* < 0.001 and ^****^*p* < 0.0001.

Supplementary Table S1 Different primers used in the study.
