# Supplementary material for: Mycobacterium smegmatis Bacteria Expressing Mycobacterium tuberculosis-Specific Rv1954A Induce Macrophage Activation and Modulate the Immune Response
Source: Front Cell Infect Microbiol. 2020 Oct 9;10:564565. doi: 10.3389/fcimb.2020.564565 (PMC7583720; doi:10.3389/fcimb.2020.564565)
Supplement: Supplementary Figure S1 — M.tb Rv1954A revealed its antigenic potential through in silico analysis. (A) B-cell and T cell epitope prediction in Rv1954A by IEDB tool (http://tools.immuneepitope.org). (B) Anchor server was used to predict presence of protein binding sites in disordered regions (https://iupred2a.elte.hu). [file Data_Sheet_1.pdf]

(A)

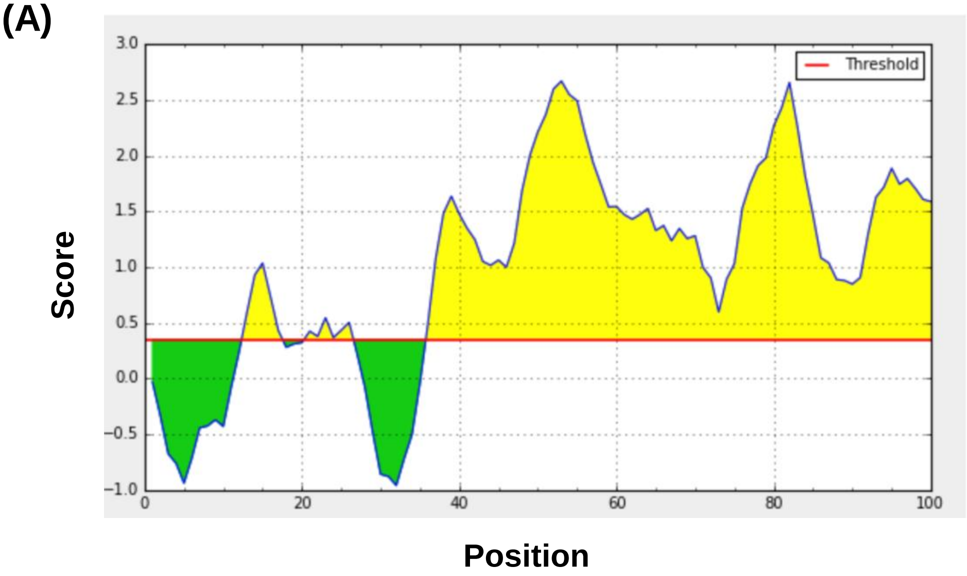

CD4 Immunogenicity prediction results

Number of proteins: 1
Number of 15mer (overlapping 10mer): 18
Threshold : 70.0%
Method : combined

Download result

Citations

Protein Number	Protein Description	Peptide	Start	End	Combined Score	Immunogenicity Score	Peptide core	Median Percentile Rank (7-allele)	HLA-DRB1:03:01	HLA-DRB1:07:01	HLA-DRB1:15:01	HLA-DRB3:01:01	HLA-DRB3:02:02	HLA-DRB4:01:01	HLA-DRB5:01:01
1	sp|P0CV86|Y954A_MYCTU Uncharacterized protein Rv1954A OS=Mycobacterium tuberculosis (strain ATCC 25618 / H37Rv) OX=83332 GN=Rv1954A PE=2 SV=1	CTPGVFRATAGGMPV	16	30	51.16956	91.9239	GVFRATAGG	24.0	43.0	16.0	48.0	24.0	12.0	70.0	23.0
1	sp|P0CV86|Y954A_MYCTU Uncharacterized protein Rv1954A OS=Mycobacterium tuberculosis (strain ATCC 25618 / H37Rv) OX=83332 GN=Rv1954A PE=2 SV=1	KWVNVDPTKNKSGRG	86	100	64.61624	94.0406	VNVDPTKNK	45.0	16.0	57.0	75.0	45.0	34.0	92.0	25.0

(B)

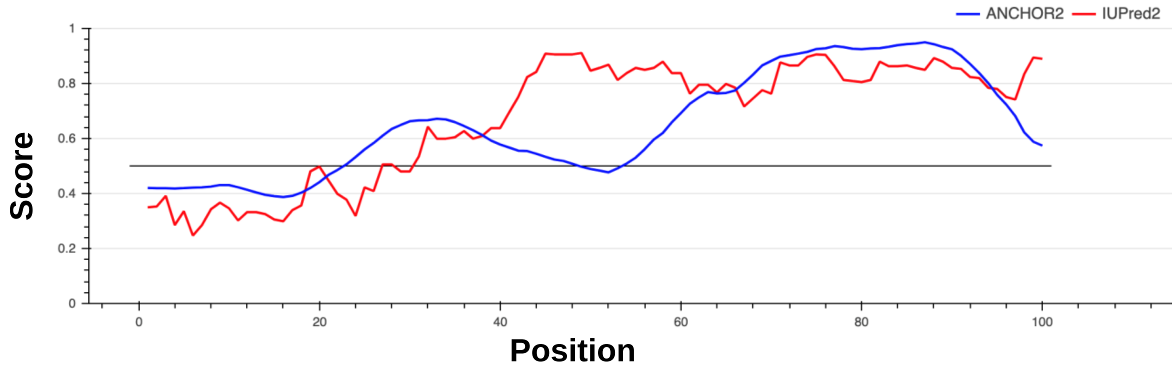

Figure S1: *M.tb* Rv1954A revealed its antigenic potential through *in silico* analysis. (A) B-cell and T cell epitope prediction in Rv1954A by IEDB tool (http://tools.immuneepitope.org). (B) Anchor server was used to predict presence of protein binding sites in disordered regions (https://iupred2a.elte.hu).

(A)

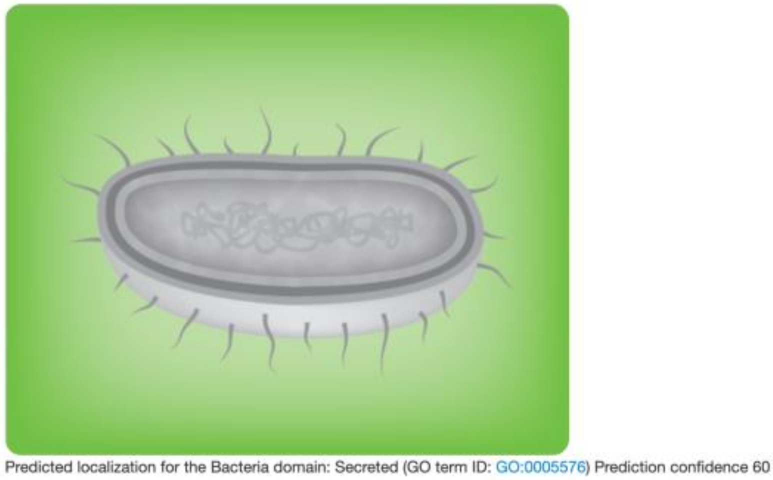

(B)

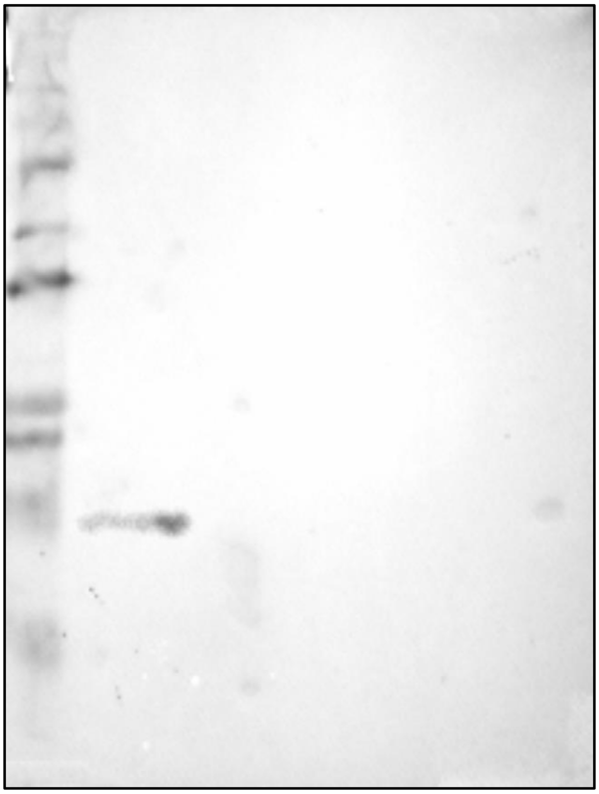

Figure S2: Secretory nature of Rv1954A is revealed through *in silico* and western blot analysis. (A) Localization prediction of Rv1954AA by PredictProtein tool (https://www.predictprotein.org/) depicting its secretory nature. **(**B**)** Western blot image depicting the expression of Rv1954A in CFP of *Mycobacterium tuberculosis* H_{37}Rv. A distinct band of 10kDa is observed in CFP of *M. tuberculosis* H37Rv but not in the CFP of BCG. (WCL: Whole Cell Lysate; CFP: Culture Filtrate Protein; M: marker).

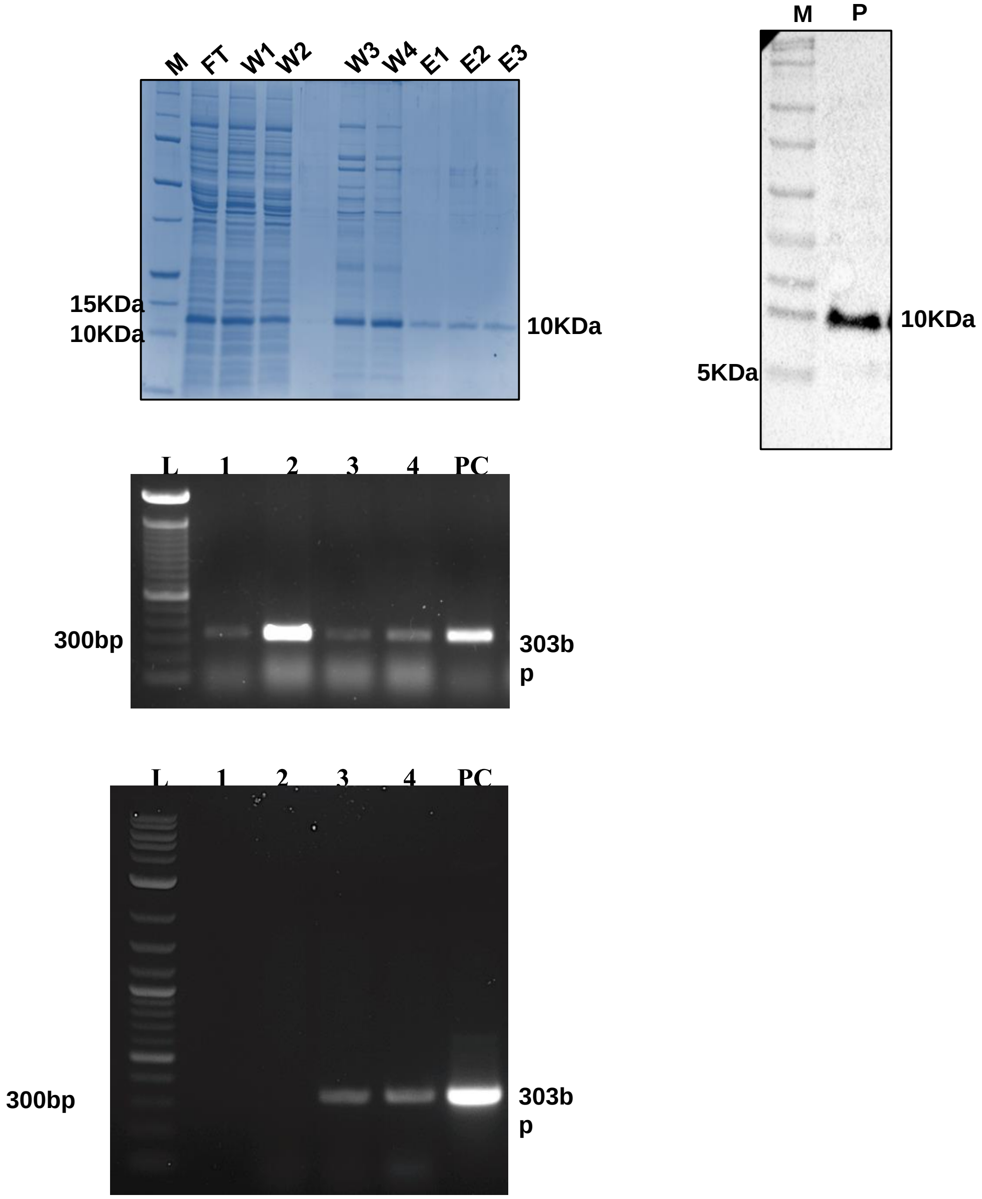

Figure S3: Expression and construction of Rv1954A knock-in *M.smegmatis* (A) SDS-PAGE image depicting the elution of recombinant *M.tb* Rv1507A. A distinct band is observed at 10KDa (M: marker; FT: flow through; W1: Wash1; W2: Wash 2; W3: Wash 3; W4: Wash 4 E1-E3: Elutions). (**B**) Confirmation of recombinant *M.tb Rv*1954A using western blot. (**C**) Molecular characterization of pST_Ki_Rv1954A knock-in *M.smegmatis*. Confirmation of Sub-cloning of Rv1507A in pST-Ki expression vector using colony PCR (PC: positive control; L: DNA ladder; 1-4: colony). **(D)** Confirmation of pST-Ki_Rv1507A in knock-in *M.smegmatis* using colony PCR. (PC: positive control; L: DNA ladder; 1-4: Colonies 1-4).

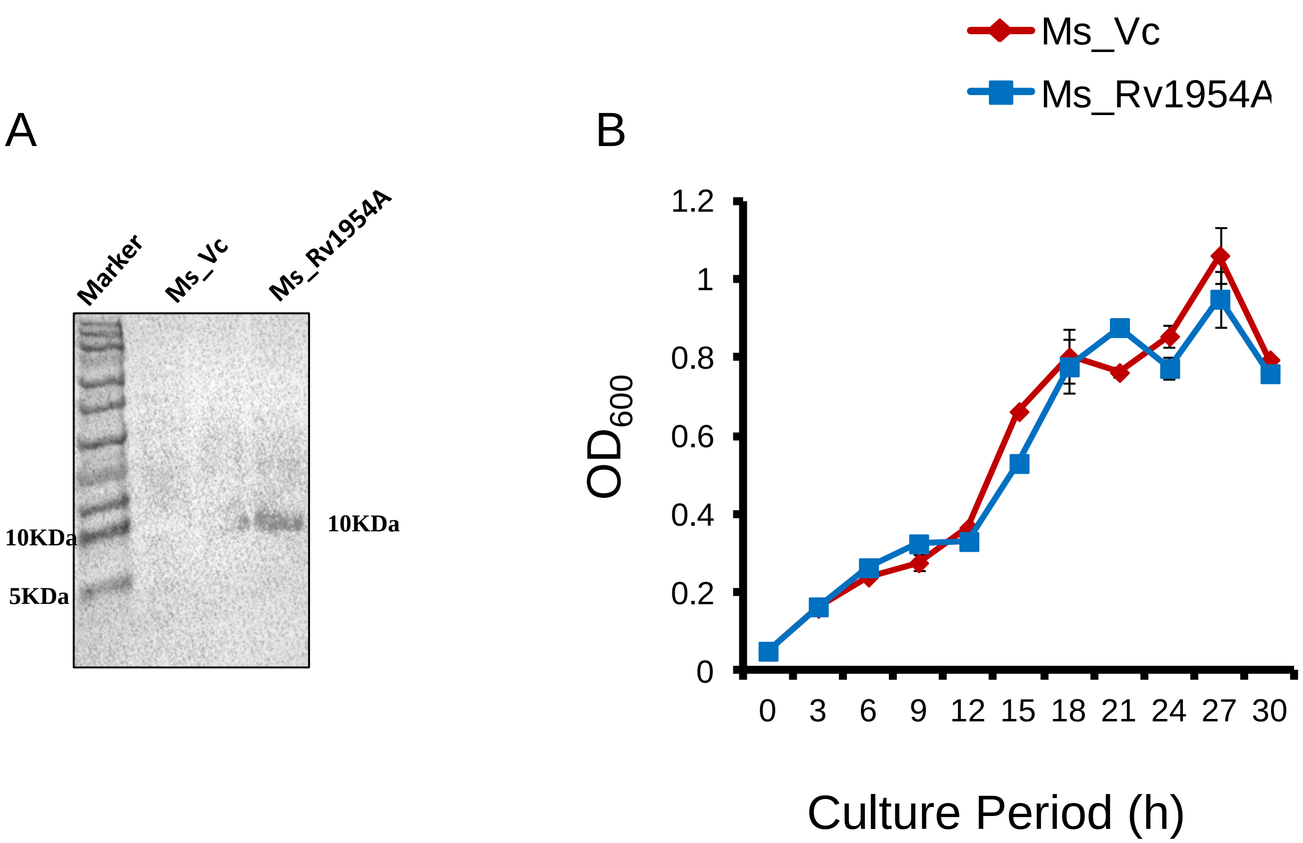

Figure S4: Rv1954A of knock-in *M. smegmatis* Ms_Rv1954A expresses Rv1954A. (A) Western blot confirmation of *M.tb* 1954A using in-house specific polyclonal antibody raised in rabbit. Note the presence of a band corresponding to 10KDa in Ms_Rv1954A lane. (B) Growth curve of Ms_Rv1954A as compared to vector control Ms_Vc. Statistical significance was determined with two-way ANOVA. Note the absence of any significant difference in terms of growth kinetics between Ms_Rv1954A and Ms_Vc.

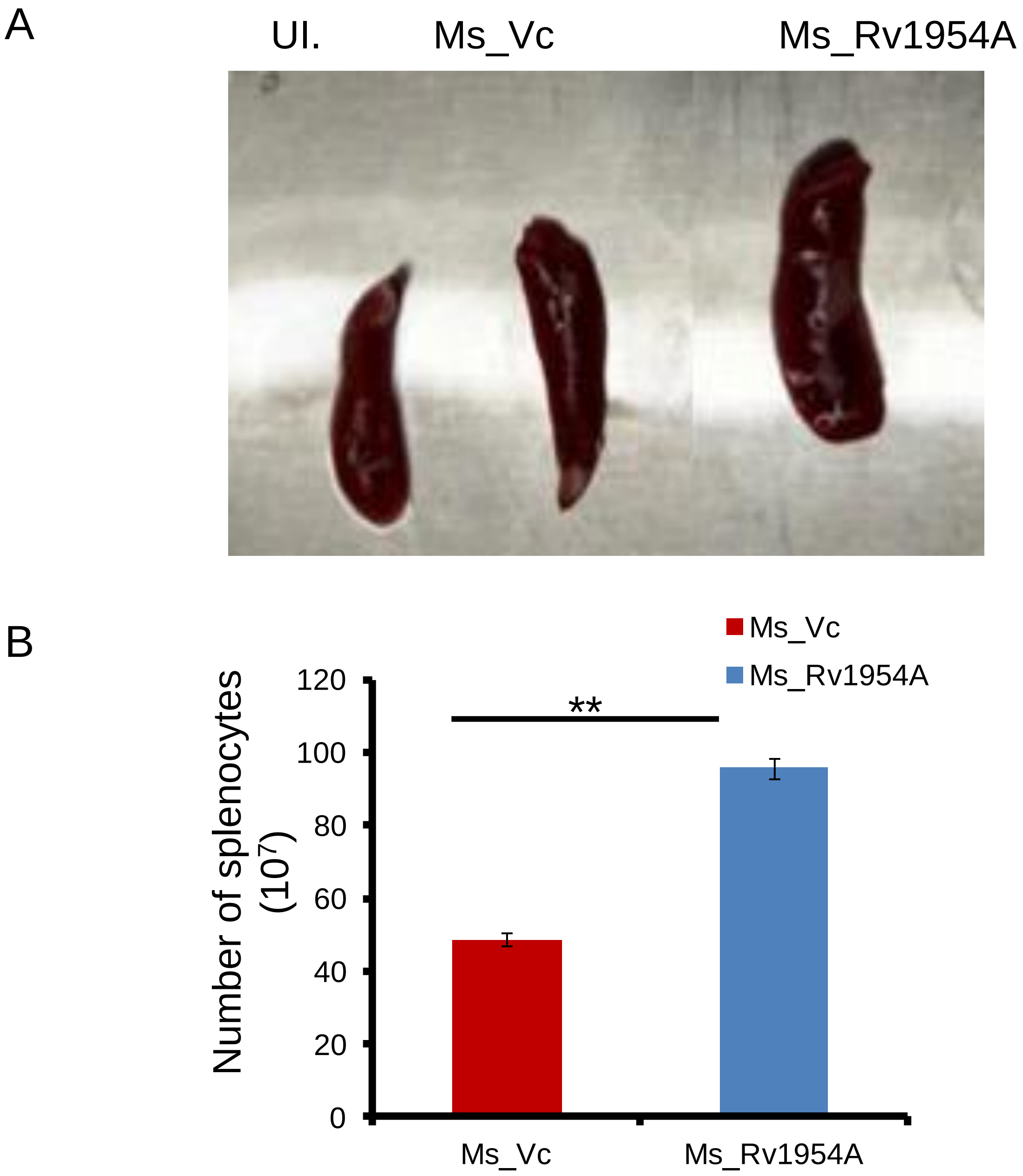

Figure S5: Splenomegaly and increased number of splenocytes is caused by Ms_Rv1954A. BALB/c mice (n=6) that were injected with either PBS (uninfected) or Ms_Vc (1×10^7) or Ms_Rv1954A (1×10^7). Spleen was recovered after one month. (A) Representative picture depicting splenomegaly in the mice infected with Ms_Rv1954A as compared to Ms_Vc. (B) The number of splenocytes was counted after making single cell suspension using trypan blue exclusion method. Representative data show the number of splenocytes as mean ± SEM. Statistical significance was determined with one tailed Mann-Whitney test. *p* value of <0.05 was considered significant, ***p* < 0.01.

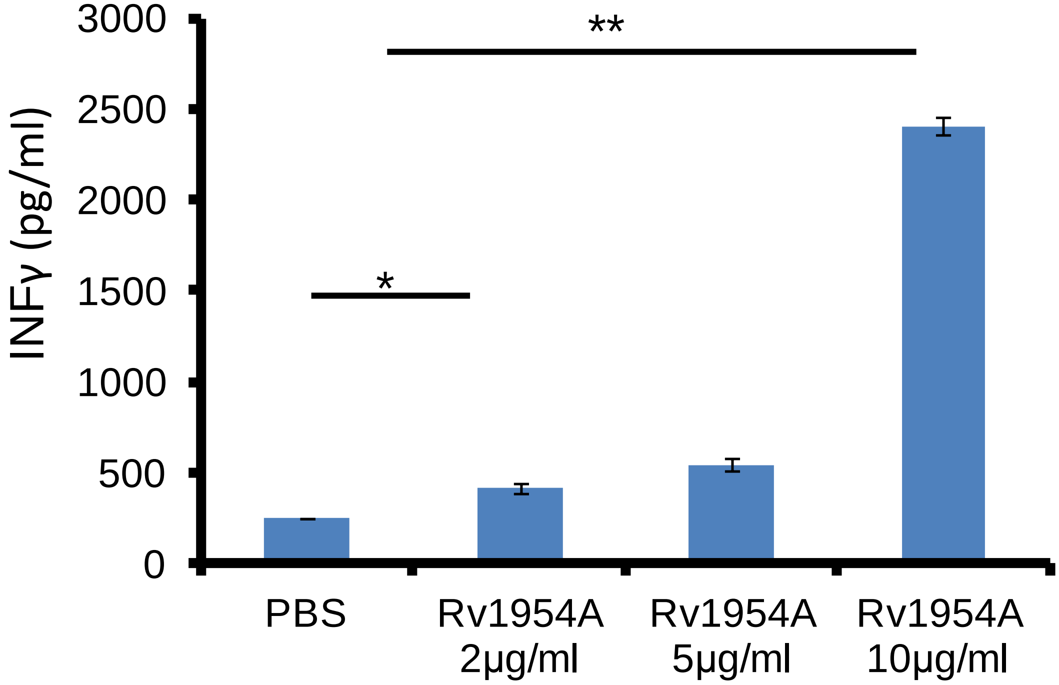

Figure S6: Induction of secretion of IFN-γ from re-stimulated splenocytes by Rv1954A. BALB/c mice (n=5) were immunized with purified recombinant Rv1954A proteins (10 µg/ml). Splenocytes (1×10^6) isolated from mice were cultured in absence or presence of Rv1954A protein (2, 5, 10 µg/ml) for 48 h and the levels of IFN-γ were estimated by ELISA. Representative data show IFN-γ secretion as mean ± SEM. Statistical significance was determined with one tailed Mann-Whitney test. *p* value of <0.05 was considered significant, *p<0.05 and ** *p* < 0.01.

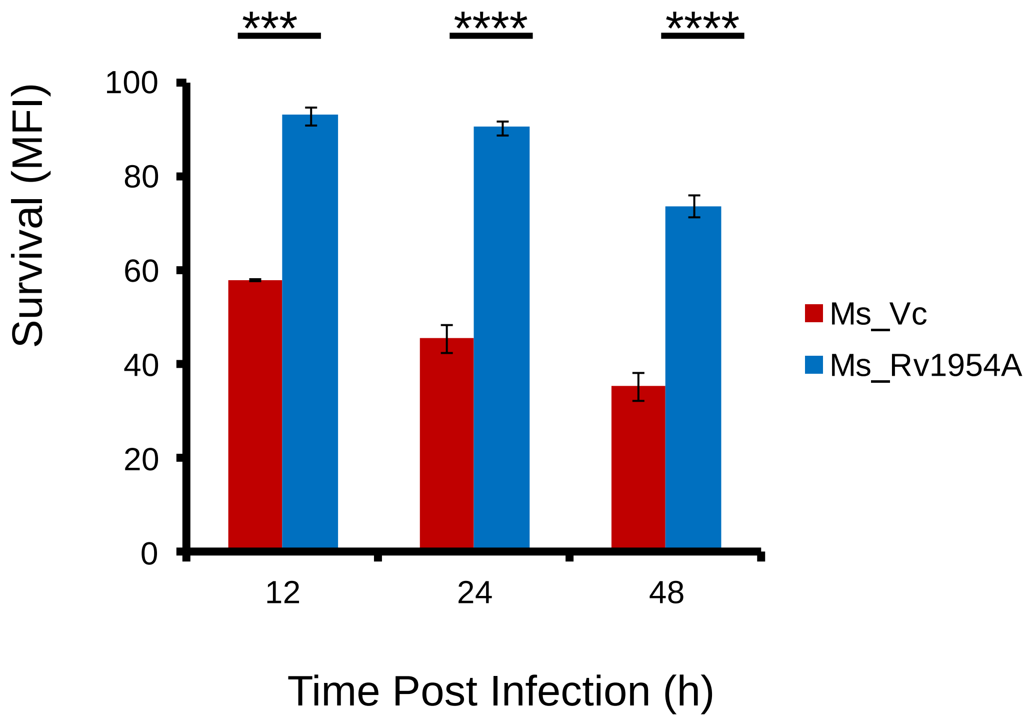

Figure S7: Increased survival of Ms_Rv1954A in infected macrophages. RAW264.7 cells were co-cultured with SYTO-9 stained Ms_Rv1954A or Ms_Vc at MOI of 10:1. The uptake of Ms_Vc and Ms_Rv1954A cells within RAW264.7 macrophage cells was assessed by flowcytometer after 12h, 24h and 48h. Representative data from three experiments show mean fluorescent intensity (MFI) of fluorescently tagged viable Ms_Vc and Ms_Rv1954A as mean± SEM. Statistical significance was determined with two-way ANOVA. *p* value of <0.05 was considered significant, ***p< 0.001 and ****$p < 0.0001$.

Supplementary Table 1: Different primers used in the study

Primer	Sequence	Restriction enzyme site
Rv1954AF (P1)	CCGGAATTCATGGCCAGGGGCAGGGTC	EcoRI
Rv1954AR (P2)	AAAGCTCGAGTCAGCCGCGGCCGCTCTTGTT	XhoI
